# The Relationship between Resiliency, Psychological Empowerment, and Teacher Burnout across Different Genders: A Psychological Network Analysis

**DOI:** 10.3390/bs14100878

**Published:** 2024-09-30

**Authors:** Danni Xue, Binghai Sun, Weijian Li, Xinwei Li, Weilong Xiao

**Affiliations:** 1 School of Psychology, Zhejiang Normal University, Jinhua 321004, China; xdddnnn@zjnu.edu.cn (D.X.); jky18@zjnu.cn (B.S.); xlxh@zjnu.cn (W.L.); 2 School of Education, Zhejiang Normal University, Jinhua 321004, China; 3 Occupational & Organizational Psychology and Professional Learning Research Unit, KU Leuven, 3000 Leuven, Belgium

**Keywords:** teacher burnout, resiliency, psychological empowerment, gender difference, psychological network analysis

## Abstract

Teacher burnout is one of the main reasons leading to decreased teaching performance and occupational mental health issues among teachers, drawing widespread global attention. Previous research has found that both resiliency and psychological empowerment can alleviate teacher burnout, yet there is no study simultaneously examining the relationships between resiliency, PE, and teacher burnout. Furthermore, previous studies have found gender differences in teacher burnout but have not examined the moderating effects of resiliency and psychological empowerment by gender group. Additionally, traditional analytical methods may overlook the compositional connections between these variables. To bridge this gap, we employed psychological network analysis to evaluate the psychological network of teachers with burnout across different genders. Findings indicate: (1) Female teachers exhibit a stronger link between their sense of departmental control and burnout, while male teachers show a stronger connection between solving instructional challenges and burnout. (2) Patience in male teachers’ approach to teaching may enhance connections with their environment, and mastering job-relevant skills can boost male teachers’ job happiness. (3) Female teachers’ “I feel connected to others” demonstrates higher bridge centrality. In comparison, male teachers’ “My work is vital to me” shows higher bridge centrality, indicating deeper connections with other symptom clusters. This study reveals the complex interactions among the factors of teacher burnout and investigates how gender differences influence the associations between these factors and burnout, by not only filling gaps in previous research but also offering new perspectives and strategies for understanding and intervening in teacher burnout, especially in the context of gender differences.

## 1. Introduction

Since the 1970s, burnout has evolved into a pervasive global issue, characterized by detrimental effects on individuals and organizations alike [1]. This phenomenon, as highlighted by Gabriel and Aguinis [2], exacts a heavy toll, manifesting in reduced performance, increased absenteeism, and a myriad of mental health challenges. The ramifications extend beyond the workplace, encompassing physical ailments such as coronary heart disease, as well as mental health disorders like insomnia and depression, as indicated by Salvagioni et al. [3].

Among various professions, educators, particularly teachers, stand out as highly vulnerable to burnout due to the substantial stressors inherent in their roles [4]. Managing student dynamics, navigating conflicts with parents, and juggling multifaceted responsibilities are perennial challenges faced by teachers [5], which contribute to a higher prevalence of burnout compared to other occupations [6]. Notably, the prevalence of teacher burnout (TB) varies significantly, influenced by factors such as sociodemographics, organizational dynamics within schools, and job-related stressors [7]. The consequences of TB extend far beyond the individual, impacting educational quality and institutional effectiveness. Teachers experiencing burnout exhibit diminished productivity and deliver lower-quality services [8]. Moreover, the repercussions of TB reverberate within the classroom environment, affecting the quality of interactions with students and exacerbating conflicts. Teachers grappling with burnout find it challenging to forge meaningful connections with their students, leading to a deterioration in the caliber of student–teacher relationships [9]. This challenge is particularly pronounced in secondary school settings, where elevated job pressures exacerbate the symptoms of burnout, characterized by emotional exhaustion, depersonalization, and diminished feelings of accomplishment [10]. In light of these multifaceted challenges, addressing the root causes of TB and implementing effective interventions represent imperative tasks for educators and educational institutions alike.

Within the academic realm, a unified consensus regarding the causes of burnout remains elusive. This complexity arises from the diverse array of professions affected by burnout, leading researchers to posit a range of contributing factors. Among these, long working hours and a dearth of colleague support are commonly cited as catalysts for burnout’s proliferation [8]. Koutsimani et al. [11] established a significant correlation between burnout and occupational mental health issues, such as depression (*r* = 0.520) and anxiety (*r* = 0.460), underscoring the intricate interplay between psychological well-being and burnout.

The causes of burnout are multifaceted. Positive traits or feelings at the individual level, which directly influence how a person handles difficult work situations, may also be influenced by gender. However, despite extensive research by scholars, there remains a significant gap in understanding the complex interplay of factors that lead to TB.

### 1.1. The Effects of Resiliency on TB

Burnout is a multi-faceted phenomenon that involves complex interactions between individual, environmental, and cultural factors. In this context, resilience, as the ability to recover quickly from adversity, becomes particularly important [12]. In the field of education, teacher resilience is especially critical as it not only affects teachers’ ability to maintain their enthusiasm for education but also relates to their ability to cope with job uncertainty [13]. Research indicates that individual resilience is closely associated with reducing the levels of burnout and compassion fatigue [14]. Resilience factors such as adaptability and self-control are key to preventing burnout [15]. Specifically in the field of education, teacher resilience helps alleviate work stress and feelings of exhaustion [16] and has a significant preventive effect on burnout and intention to quit [17]. There is a negative correlation between psychological resilience and burnout [18], meaning that the stronger the resilience, the lower the likelihood of burnout. Hence, enhancing teacher resilience is an effective strategy for preventing and alleviating burnout.

There is a positive correlation between teachers’ high levels of burnout and their high levels of negative emotions toward students and various psychopathological symptoms, while higher perceived resilience is associated with lower levels of negative emotions, burnout, and psychopathological symptoms [19]. In specific social and educational environments, teachers’ resilience can resist negative job demands, increase positive job resources, and ultimately enhance teachers’ well-being and job performance [20]. Additionally, teacher resilience is a potential concept that is centered on the environment and oriented toward the process [21]. It is influenced not only by individual traits such as altruistic motivation and high self-efficacy but also by environmental support from school management, colleague relationships, and student groups [22].

### 1.2. The Effects of Psychological Empowerment on TB

Psychological empowerment (PE), as defined by Spreitzer [23], encompasses a range of cognitive experiences that individuals undergo, resulting in a sense of meaning, competence, impact, and self-determination. This concept has been found to play a significant role in combating burnout. In particular, PE has been shown to effectively reduce the risk of experiencing burnout, as demonstrated in the implementation of transformational leadership practices. By fostering employees’ PE, leaders can help alleviate the occurrence of burnout [24]. For educators, PE serves as a potent tool to combat the unique challenges and psychological exhaustion prevalent in the education industry. Extensive research has consistently shown a negative correlation between PE and TB [25,26]. This relationship is especially pronounced among rural teachers, where PE is significantly associated with reduced feelings of job burnout [27].

To effectively support teachers and alleviate their burnout, it is crucial to design and implement well-planned PE programs. These programs are designed to meet the fundamental psychological needs of teachers, thereby contributing to a reduction in their sense of professional exhaustion [28]. In addition, research has highlighted the interplay between resilience and PE in the context of burnout. Both resilience and PE have been found to have significant “net effects” on job burnout, with PE partially mediating this relationship [29]. This suggests that cultivating PE, alongside building resilience, is crucial in preventing and managing burnout effectively.

### 1.3. Gender Differences in TB

Although the research results are not consistent, numerous studies have indicated significant gender differences in TB [30,31]. On one hand, several studies have shown that female teachers are more likely to experience burnout [32,33,34]. Additionally, female teachers report higher levels of trait anxiety, which may further induce job burnout [35]. On the other hand, some studies suggest that male teachers have higher levels of burnout [36,37]. Scholars acknowledge a noticeable gap in the literature regarding the role of gender in TB [38]. Especially, traditional analytical approaches lack compositional analysis and struggle to adjudicate crucial drivers within complex systems.

While resiliency and PE are known to be essential factors affecting TB, whether there are gender differences in these interacting systems remains unexplored. A few studies have revealed that the resilience teachers possess is also influenced by gender disparities [39,40]. Similarly, some studies have shown that teachers’ PE differs across genders [41,42]. Despite the research indicating that teachers’ resiliency is affected by gender inequalities and that there are differences in PE among teachers of distinct genders, there is a lack of research investigating the role of gender in the relationship between resiliency, PE, and TB. Therefore, examining gender differences is crucial for comprehensively understanding how teachers are using resiliency and PE to address teaching challenges and for developing more effective support strategies and interventions tailored to binary genders.

### 1.4. Psychological Network Analysis

Burnout is a significant issue in the human services industry, and research in the field of psychiatry plays a crucial role in developing effective treatment strategies [43]. Network analysis, as an advanced research tool, can reveal key aspects of psychopathology, including the centrality of symptoms and their interactions [44]. Compared to traditional statistical methods, the advantages of psychological network analysis lie in its ability to visualize and interpret relational data, revealing key nodes and community structures in the data through node and edge construction [45], which may be overlooked in traditional analysis. Psychological network analysis not only uncovers interactions between variables but also delves into the associations between individuals and the overall network structure [46]. This approach allows for a more precise understanding of the complexity of psychological phenomena. Specifically, psychological network analysis provides a unique perspective to intuitively understand the network relationships between symptoms or components, aiding in the identification of core nodes or bridge nodes in the “TB–Resiliency–PE” network [47]. “Node expected influence” or “node centrality” reflects the strength of connections between nodes. In contrast, “bridge expected influence” and “bridge centrality” are important indicators for assessing the role of nodes in the network [47]. “Bridge expected influence” measures information transmission between nodes, revealing key nodes connecting different communities in the psychological network, which is crucial for understanding information flow, node roles, and network functionality [48]. “Bridge centrality” measures the degree of connection between nodes in different communities, with nodes of high bridge centrality playing a core role in information transmission. Network analysis also allows for the evaluation of the accuracy and stability of network parameters through statistical methods, enhancing the credibility and interpretability of research findings [48].

### 1.5. The Current Study

To date, our understanding of the intricate relationship between resiliency, PE, and TB remains fragmented. Moreover, the potential gender disparities within this complex interplay remain largely unexplored. To shed light on these dynamics, we employed a psychological network model to elucidate the connections between these variables and unveil any gender-specific symptomatology. Our investigation sought to address two essential inquiries: firstly, whether gender differences exist in the connections between resiliency, PE, and various burnout symptoms; and secondly, whether these gender disparities extend to the bridge expected influence of symptoms within the TB–Resiliency–PE network model. While previous studies have delved into the core symptoms of TB [49], they have not systematically examined the potential drivers of TB from both resiliency and PE perspectives. Additionally, the gender variations in bridge expected influence and bridge centrality of specific symptoms within the network remain unexplored.

This study aims to provide valuable insights into the development of gender-specific differences within the TB–Resiliency–PE network. By employing network visualization techniques, we can discern and compare the differential impacts of various factors, thereby identifying those with significant influences. Building upon these premises, we hypothesize that significant gender disparities exist in both the connections of the TB–Resiliency–PE network and the bridge expected influence of symptoms. This exploration not only enhances our understanding of gender-specific manifestations of burnout but also offers actionable insights for interventions aimed at mitigating its effects.

## 2. Methods

### 2.1. Participants

The participants were in-service teachers from Zhejiang province, China. Initially, we recruited 3991 educators, then we excluded those who did not meet the criteria for burnout (burnout scale score < 24) [50]. Finally, the sample included 1729 participants, comprising 541 men and 1188 women. For male educators, the average age was 42.17 years (*SD* = 8.21), with age information missing for 3 male teachers (0.55%). The average teaching experience for male teachers was 21.93 years (*SD* = 10.58), with 467 teachers (86.32%) majoring in teaching programs and 74 teachers (13.68%) majoring in non-teaching programs. For female educators, the average age was 36.64 years (*SD* = 8.17), with age information missing for 7 female teachers (0.59%). The average teaching experience for female teachers was 15.77 years (*SD* = 10.55), with 989 teachers (83.25%) majoring in teaching programs and 199 teachers (16.75%) majoring in non-teaching programs.

Despite the typical characteristic of psychological research having a relatively small sample size [51], we conducted a priori power analysis for the sample sizes of male and female teachers to ensure an adequate sample size before formal analysis [46,52]. The results showed that for male teachers, the correlation between the “true” network and the estimated network (edge weights or centrality estimates) was greater than 0.90, the sensitivity (true-positive rate) was greater than 0.65, and the specificity (true-negative rate) was greater than 0.70. For female teachers, the correlation was greater than 0.95, sensitivity (true-positive rate) was greater than 0.75, and specificity (true-negative rate) was greater than 0.75 (further details of the power analysis graph can be found in the Supplementary Materials). These results approached the acceptable results of previous power analyses in psychological network analysis (correlation > 0.80, sensitivity > 0.70, and specificity > 0.70) [46].

### 2.2. Measures

As part of a large study on teachers’ professional environment and mental health, a 28-item anonymous questionnaire was used, with teachers taking approximately 7–10 min to complete this section. The study focuses on TB, resiliency, and PE. In addition, we collected demographic information relevant to our research questions from the participants. The recorded information includes the city of the teacher, age, gender, professional title, years of teaching experience, major, grade and subject taught, and academic qualifications.

### 2.3. TB

To measure burnout, the Professional Quality of Life Scale (ProQOL) subscale—which was created by Stamm [50] and amended by Chen and Wang [53]—was employed. This measure has also been used with Chinese teachers in the past, with positive results for high validity and reliability [54,55]. With a 5-point Likert scoring system ranging from 1 (never) to 5 (very often), 10 items make up this subscale. Items (1, 2, 5, 6, 10) like “I am happy”, “I feel connected to others”, “I have beliefs that sustain me”, “I am the person I always wanted to be”, “I am a very caring person”, were among the half of the items that were assessed using reverse scoring. The internal consistency coefficient for this subscale was good (*α* = 0.75) [53]. The Cronbach’s *α* coefficient of the sample in this study was 0.88.

### 2.4. Resiliency

We utilized a subscale of the Psychological Capital Scale to measure teachers’ resilience [56]. The resilience dimension of the Psychological Capital Scale comprises 6 items (e.g., “I can still work calmly after being criticized by leaders”). Particularly, the scale employs a 6-point Likert scoring system, with reverse scoring for item 2: “I’m often overwhelmed when I’m having trouble teaching.” Each statement was rated on a scale from 1 (strongly disagree) to 6 (strongly agree). High levels of validity and reliability have been documented for this scale in China [49,57]. The study revealed that the Cronbach’s alpha coefficients of the subscale ranged from 0.62 to 0.82. The Cronbach’s *α* coefficient of the subscale in this study was 0.72.

### 2.5. PE

The assessment of educators’ PE utilized the PE Scale (PES), a 5-point Likert scale developed by Spreitzer [23]. The scale’s items were rated from 1 (strongly disagree) to 5 (strongly agree). The Chinese version of the PES, compiled by Li et al. [58], was utilized in this investigation. The scale comprises 12 items across four dimensions: meaning (e.g., “What I do at work is very meaningful to me personally”), self-determination (“I have a lot of autonomy in deciding how to get my work done”), self-efficacy or competence (“I have acquired the skills needed to do my job”), and impact (e.g., “I have a lot of control over what happens in my department”). Several researchers have shown that this scale has good validity and reliability, especially when applied to Chinese populations (e.g., [59]). In this study, the Cronbach’s *α* coefficient for this scale was 0.93.

### 2.6. Procedure

The study adhered to the ethical standards outlined in the Helsinki Declaration and obtained approval from the local institution’s ethics committee before commencing data collection. Before participating in the experiment, all participants provided written informed consent to confirm their willingness to take part. Data collection began in November 2021, and participants did not receive any form of compensation, bonus, or award for their involvement. The online survey platform, operated by the reputable organization Credamo (www.credamo.com, accessed on 5 November 2021), was tailored for the Chinese context and resembled the Qualtrics online platform. Participants were assured that their responses would be treated with absolute confidentiality and used solely for scholarly research, safeguarding their privacy.

### 2.7. Statistical Analysis

Our data for the three main variables were complete. We conducted descriptive statistical tests using SPSS version 26.0 and performed psychological network estimation and accuracy measurement on the male and female teacher groups using R (version 4.3.2 in RStudio 2023.12.0+369) [46,60]. For network estimation, we employed the graphical least absolute shrinkage and selection operator (GLASSO) [61] to create a sparse network by setting minor edges to zero. To prevent overfitting in models with a large number of parameters, we utilized regularization techniques. These techniques fine-tune the hyperparameters to minimize parameter values, which can lead to the elimination of some edges.

Different λ values (a hyperparameter of GLASSO) can result in varying network configurations, and we used the default λ setting for our analysis. Additionally, we applied the extended Bayesian information criterion (EBIC) [62] to select among models with different levels of sparsity, known as EBICglasso [46]. This method allows for the incorporation of Spearman correlations [63]. While there is no automated procedure for selecting the EBIC hyperparameter (γ), a value of 0.5 is commonly recommended [46,64].

In contrast to the widely used Pearson coefficient, the Spearman coefficient is rank-based, non-parametric, and scale-invariant [65]. Similar to prior research [66], we employed the Spearman rank correlation, which avoids the disappearance of true edges, does not require data to follow a normal distribution, and is suitable for network comparison tests [60,67]. The network plots were drawn using the qgraph package [68]. By setting the layout of the nodes to “spring”, we were able to create a force-directed layout using the Fruchterman–Reingold algorithm [69] ported from the SNA package, bringing nodes with stronger correlations closer to each other.

Centrality measures are employed to quantify the importance of nodes within a network. Various types of centralities exist, each emphasizing different aspects of a node’s role within the network. For instance: Strength Centrality refers to the sum of the absolute values of the edge weights connected to a node, indicating its strong connections to many other nodes [70]. Closeness Centrality measures how close a node is to all other nodes, reflecting its ability to quickly influence others through short paths [71]. Betweenness Centrality assesses the extent to which a node lies on the shortest paths between other nodes, acting as a bridge or intermediary [72]. Expected Influence is the total of all edge weights connected to a node, which quantifies its overall connectedness regardless of edge direction [73]. In the context of psychopathology networks, strength, and expected influence are particularly valuable as they help identify the symptoms most critical for understanding the spread of symptoms [60].

Regarding Bridge Centrality metrics, including Bridge Strength and Bridge Expected Influence, these measures are used to identify nodes that connect symptoms of different psychological disorders, often referred to as bridging symptoms. Bridge Strength indicates the total connectivity of a node with symptoms from other disorders, while Bridge Expected Influence considers the sign of edge weights, reflecting the overall tendency of a node to increase activation levels in connected nodes [74]. The activation of bridging symptoms in the network model is likely to result in the development and maintenance of two symptom clusters. We estimated the bridging centrality indices using the bridge function in the R package “networktools” [75] to identify bridges between symptom clusters, offering insights into the role of symptoms in connecting different clusters [45].

In psychological networks, edges represent the connections between two nodes. These connections can be weighted to indicate the strength of the relationship or unweighted to signify merely the presence of a connection. Additionally, edges can be directed, showing the influence of one node on another, or undirected, indicating a mutual relationship. In the networks discussed in this study, edges reflect the strength of associations between nodes, which are estimated using (partial) correlations [76].

We conducted differential testing on the edge weights and used the nonparametric bootstrap procedure from the R software (version 4.3.2 in RStudio 2023.12.0+369) package “bootnet” to determine the stability and accuracy of the network. During this process, we plotted the 95% confidence intervals (CIs) for each edge using 1000 samples. Using case-dropping bootstraps, the four centrality indices’ stability as well as the bridging variation in strength and expected influence were evaluated [60]. In particular, between 10% and 75% of the samples were eliminated, and the remaining data were utilized to estimate the network model. As to Epskamp et al. [60], there should be a correlation stability (CS) coefficient of at least 0.25 between the original model and the subset models.

Gender differences in the TB–Resiliency–PE network were examined using the permutation test from the R package “Network Comparison Test” [67]. Four indicators were examined: each edge’s strength, the global strength (the sum of the weighted absolute values of all the network’s edges), and the expected influence of nodes and bridges [67,73]. The Supplementary Materials contain the outcomes for these metrics.

## 3. Results

### 3.1. Results of Descriptive the Sample

The descriptive statistics of the items and scales are shown in Table 1. While we recognize the concern regarding Type I error inflation due to multiple comparisons, we have carefully considered this issue. To mitigate the risk, we applied appropriate corrections (e.g., Bonferroni adjustment). There is no significant difference in the level of burnout between male and female teachers. In terms of burnout, male teachers have significantly higher scores on Burnout 3, 4, and 9 compared to female teachers, while female teachers have significantly higher scores on Burnout 5 compared to male teachers. In terms of PE, male teachers have significantly higher scores on PE7, as well as PE10–12, compared to female teachers. In terms of resiliency, male teachers have significantly higher scores on Resiliency 3 compared to female teachers.

### 3.2. Gender Difference in Network Estimation

**Figure 1:**
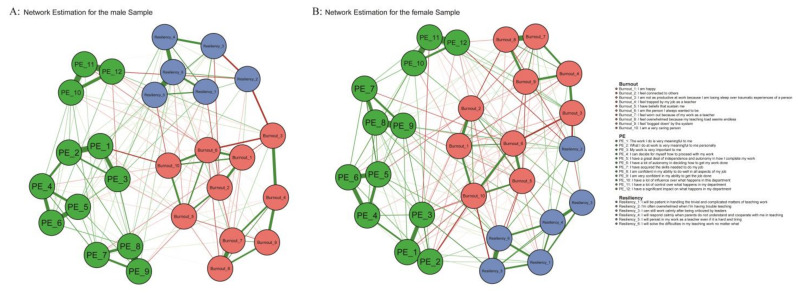
(**A**) The network estimation of TB–Resiliency–PE for male teachers and (**B**) the network estimation of TB–Resiliency–PE for female teachers. Note. Positive correlations are represented by blue edges, while negative correlations are represented by red edges. The thickness of the edges indicates the strength of the correlation. PE = Psychological Empowerment.

**Figure 2:**
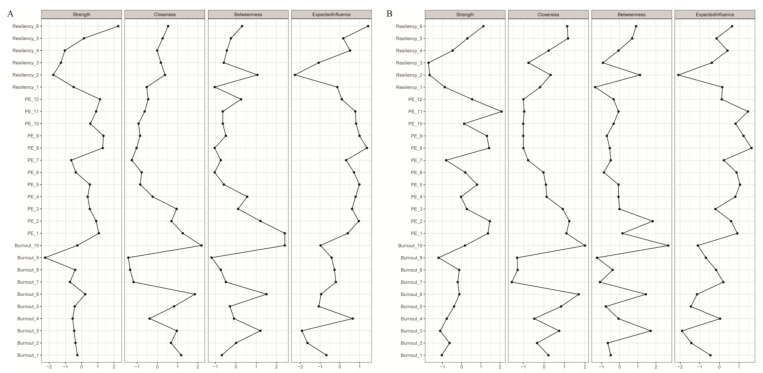
(**A**) centrality indices of male teachers and (**B**) centrality indices of female teachers. All measures are Z-standardized. PE = Psychological Empowerment.

The network estimation graphs for male and female teachers are represented in Figure 1A and Figure 1B, respectively, and the centrality indices are depicted in Figure 2A,B. In the network of male teachers, out of 378 possible edges, there are 149 non-zero edges, whereas in the network of female teachers, there are 170 non-zero edges. There are also some differences in the centrality indices of the nodes in the two graphs. For male teachers, resilience 6 “I will solve the difficulties in my teaching work no matter what” has the highest strength and expected influence centrality, followed by PE 8 “I am confident in my ability to do well in all aspects of my job.” Burnout 10 “I am a very caring person” has the highest closeness and betweenness centrality, followed by Burnout 6 “I am the person I always wanted to be.” For female teachers, PE 11 “I have a lot of control over what happens in my department” has the highest strength and expected influence centrality, followed by PE 8. The symptoms with the highest closeness and betweenness centrality are the same as those for male teachers.

### 3.3. Gender Difference in Network Stability and Accuracy

The bootstrap procedure for edge weights was implemented at 95% CIs to ensure network accuracy. The outcomes demonstrated that the bootstrap means of edge weights were close to the sample edge weights and the bootstrap CIs for most of the sample edge weights are small. These results showed how accurate the network was (see Supplementary Materials Sf1 and Sf2 (female) and Sm1 and Sm2 (male)). In order to further quantify the stability of the network, we implemented the case-dropping bootstrap procedure and also included the 95% CIs. The average correlations did not sharply decrease as the sample size decreased, and the stability of the female teacher group was slightly better than that of the male group, as shown in the results. We calculated the CS coefficients, which for male teachers were strength = 0.67, expected influence = 0.75, closeness = 0.44, and betweenness = 0.28. For female teachers, strength/expected influence = 0.75, closeness = 0.52, and betweenness = 0.36. Both sets of results indicate that our network has good stability. For more detailed information on the results of the network stability analysis, please refer to the Supplementary Materials.

### 3.4. Gender Difference in Edges and Centrality

The edge invariance test in the TB–Resiliency–PE network revealed that there were three edges showing gender differences (*p* < 0.01). These were (1) the edge between Resiliency 1 “I will be patient in handling the trivial and complicated matters of teaching work” and Burnout 2 “I feel connected to others”; (2) the edge between Resiliency 3 “I can still work calmly after being criticized by leaders” and PE 3 “My work is very important to me”; and (3) the edge between Burnout 1 “I am happy” and PE 7 “I have acquired the skills needed to do my job.” Additionally, there were 12 edges displaying gender differences (*p* < 0.05). Furthermore, the test for strength centrality in the male and female teacher networks indicated gender differences in the strength of two nodes (*p* < 0.05). These were: Burnout 1 and PE 11 “I have a lot of control over what happens in my department.” Moreover, there were significant gender differences in the expected influence of nodes on Resiliency 3 and 6, Burnout 4, and PE 3 and 11. For more details, please refer to the Supplementary Materials.

### 3.5. Gender Difference in Bridge’ Centrality

**Figure 3:**
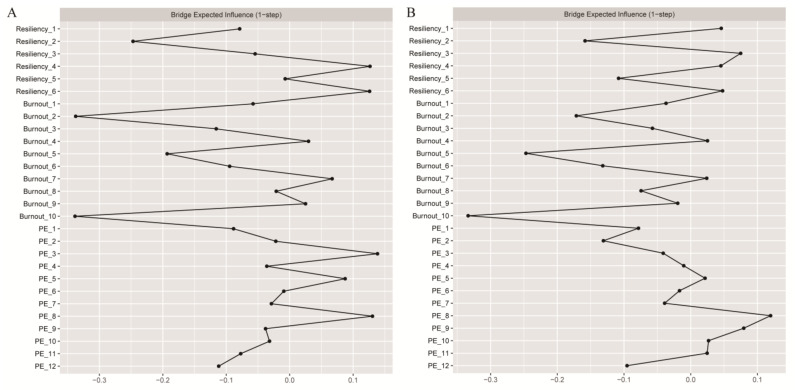
(**A**) Bridge expected influence of male teacher networks and (**B**) bridge expected influence of female teacher networks. PE = Psychological Empowerment.

The centrality invariance test revealed that there were two significant gender differences (*p* < 0.05) in the bridge expected influence in the TB–Resiliency–PE network. The bridge centrality of Burnout 2 “I feel connected to others” was stronger for female teachers compared to male teachers. However, the bridge centrality of PE 3 “My work is very important to me” was stronger for male teachers compared to female teachers (see the last part of the Supplementary Materials). Figure 3A,B display the bridge expected influence of each node in both male and female teachers. For all teachers, the CS coefficient of bridge strength was 0.67, and the bridge expected influences were both 0.75, indicating a high level of stability.

## 4. Discussion

The innovation inherent in this study resides in its utilization of network analysis methodologies to elucidate the manifestation of teacher burnout symptoms within binary gender categories. Additionally, the analysis of scale scores revealed no statistically significant differences in total burnout levels between male and female teachers. However, network analysis uncovered differences in the central and bridging symptoms within the two teacher groups. These symptoms serve distinct functions within the symptom networks of male and female educators. Central symptoms are those with a high degree of connectivity within their own cluster, thereby playing a critical role in maintaining the overall structure of the network. In contrast, bridging symptoms link different symptom clusters, connecting otherwise disparate communities within the network. Both central and bridging symptoms are pivotal to the integrity and functionality of the teacher symptom network.

### 4.1. Gender Differences in Core Network Symptoms

This study reveals that there are similarities in the core symptoms of burnout networks between male and female teachers, but differences in the influence of crucial nodes in the network. Specifically, PE 11 “I have a lot of control over what happens in my department” has a greater impact on the network of female teachers, while Resiliency 6 “I will solve the difficulties in my teaching work no matter what” has a greater impact on the network of male teachers. Previous studies have also suggested that burnout in female teachers is related to the social psychological work environment (i.e., lack of work control) [77]; male teachers may focus on solving problems themselves [78], and their sense of internal control may be stronger than that of female teachers [79]. It is worth noting that PE 8 “I am confident in my ability to do well in all aspects of my job” is important for both male and female teachers, reflecting the self-efficacy in the work aspect of teachers. Teacher self-efficacy is positively correlated with classroom quality and teacher mental health, negatively correlated with burnout factors [80], and plays an important role in influencing well-being in the work environment [81], improving job satisfaction and student academic performance [82]. This suggests that we can prevent burnout by increasing teachers’ confidence in doing their job well.

In the group of teachers, the most prominent symptoms of burnout are Burnout 10 “I am a very caring person” and Burnout 6 “I am the person I always wanted to be.” Considering the reverse scoring, it means that burnt-out teachers find it difficult to show high levels of care and are unable to achieve their ideal self-image. Figley [83] points out that burnout is one of the inherent “costs of caring” in helping professions. Similar to other helping professions, teachers also bear a considerable degree of the cost of caring, as evidenced in the study by Brown and Biddle [84]. The intimate interaction between teachers and students is integral, but it is also one of the reasons for teacher fatigue and emotional downturn [85]. Also, if teachers are dissatisfied with their educational work and feel unable to achieve their ideals, they may feel frustrated. For example, Pedditzi et al. [86] found that secondary school teachers experience emotional exhaustion and a decrease in personal achievement due to burnout.

### 4.2. Gender Differences in the Connections between Symptoms

The significant differences found between male and female teachers in the teacher network—3 edges (*p* < 0.01) and 12 edges (*p* < 0.05)—indicate variations in the connections of burnout, resilience, and PE. For example, male teachers exhibited a stronger negative correlation between Resilience 1 (“I will be patient in handling the trivial and complicated matters of teaching work”) and Burnout 2 (“I feel connected to others” [reverse scoring]). Prior research suggests that female teachers may often be perceived as more patient and supportive of students [87,88,89], and studies like Karayaman’s [90] highlight the importance of patience and resilience in organizational settings. However, these tendencies may be culturally specific, and it is essential to acknowledge that perceptions and behaviors related to patience in teaching can vary across different contexts. Encouraging patience and attentiveness in male teachers may improve their relationships with students and enhance their connection to the school, potentially reducing burnout among male teachers.

Second, the observed stronger positive correlation between Resiliency 3 “I can still work calmly after being criticized by leaders,” and PE 3 “My work is very important to me,” within the male teacher network suggests a significant linkage between emotional resilience and work engagement, particularly among male educators. This robust correlation implies that for male teachers, a high level of job engagement—characterized by the belief that their work holds considerable importance—is closely associated with an enhanced ability to remain composed and continue performing effectively even after receiving criticism from superiors.

One plausible explanation for this phenomenon is rooted in the existing literature on teacher engagement and resilience [91]. Teachers who are highly engaged often exhibit a strong sense of identification with, and satisfaction in, their professional roles. This deep-seated commitment enables them to navigate challenges and process negative feedback more effectively. Specifically, these teachers are more likely to interpret criticism as constructive, viewing it as an opportunity for professional growth rather than as a personal affront. Consequently, the pronounced positive correlation between resilience and engagement among male teachers may reflect their capacity to positively integrate feedback, a process that is likely reinforced by their intrinsic motivation and strong attachment to their work.

Lastly, the negative correlation between Burnout 1 “I am happy” (reverse scoring), and PE 7 “I have acquired the skills needed to do my job” in the male teacher network is also stronger than in the female teacher network. In other words, they may be more inclined to view mastery of job skills as a key factor in achieving job satisfaction. On the one hand, there are gender differences in job attribute preferences, with men placing more emphasis on job skills than women [92], especially men in non-traditional professions such as elementary school teachers [93]. On the other hand, teachers’ skills and efficiency will change over time and require continuous and concentrated efforts from teachers, leaders, and school management [94]. Effective skill application models combined with comprehensive approaches can greatly improve teachers’ professional level and abilities [95], and teachers’ professional development can improve classroom teaching, which is directly related to the improvement of classroom teaching and student success [96]. In terms of the external educational environment, schools should endeavor to develop the professional skills of teachers (especially male teachers) to increase their job satisfaction and reduce burnout.

### 4.3. Gender Differences in Bridge Centrality of Network

Our results indicate that female teachers have stronger bridge centrality in the “TB–Resiliency–PE” network, specifically with Burnout 2 “I feel connected to others.” Bridge centrality measures the extent to which a node acts as a critical link between different parts of the network. A higher bridge centrality value suggests that the node plays a pivotal role in connecting otherwise less-connected components of the network.

Research highlights that the need for relatedness is a fundamental human need, encompassing the desire to form interpersonal relationships and connect with others [97]. Studies have often linked women’s identities to their relational connections [98], and in education, “caring” is frequently associated with female roles [99]. For female educators, their relationships with others can improve their work experience and foster autonomy, leading to positive outcomes [100]. Strengthening connections with students, colleagues, and the community could be an effective way to reduce burnout among female teachers.

In contrast, male teachers exhibit higher bridge centrality for PE 3 “My work is very important to me.” This suggests that the perceived importance of their work plays a key role in linking different aspects of their experiences. Male teachers might place greater emphasis on professional identity and self-actualization, which could have a significant impact on their burnout levels. Professional identity is a key motivator for teachers [101], and research suggests that teachers’ professional identity is constantly negotiated in response to the changing educational landscape [102]. Understanding these gender differences in how work importance is perceived can provide valuable insights for alleviating burnout and supporting professional growth, particularly for male teachers. Further research could explore strategies to enhance the perceived importance of work among teachers to mitigate burnout and promote job satisfaction.

### 4.4. Implications

Unlike previous studies that investigated the relationship between gender and TB (e.g., [102,103]), we utilized a psychological network analysis approach to explore the relationship between resiliency and PE on TB and the gender differences therein. Furthermore, we examined the bridge centrality of network nodes, providing researchers and other educational practitioners with a new perspective to identify and emphasize core risk factors. In educational settings such as schools, gender-specific targeted measures can be taken to prevent and intervene in teacher burnout. It is possible to not only improve teaching performance but also enhance teacher well-being.

Specifically, for female teachers, our findings indicate that external factors exert a more substantial influence on them. Consequently, the primary intervention strategies should include (1) Enhancing female teachers’ sense of control within their departments. This could be achieved by providing targeted training for female educators, promoting gender equity in school leadership positions, and raising societal awareness regarding the acceptance of women in leadership roles [104,105,106,107]. These measures could effectively bolster women’s perceived control over their departmental roles, thereby mitigating teacher burnout. (2) Strengthening the connections female teachers have with their surroundings, such as fostering stronger relationships between students and teachers [108], could also contribute to creating a more supportive environment, ultimately reducing burnout.

Male teachers’ burnout is influenced by multiple factors related to internal qualities and external environments. Prior studies have indicated that resiliency helps educators deal with obstacles by allowing them to adjust, react, and recover from setbacks. It is possible to acquire, hone, and reinforce this capacity [109]. Thus, the main intervention strategies are as follows (1) Efforts should be directed toward cultivating male teachers’ abilities to solve teaching challenges and enhancing their teaching skills. Considering that previous studies have shown that male teachers demonstrate higher proficiency in using digital technology for peer interaction, strengthening assessment processes, and promoting student digital literacy [110], future research could further enhance this area of strength. (2) Patience levels when facing complex teaching tasks should be increased among male teachers. Meriç [111] noted in their study that, generally, teachers with more experience excel in patience. Therefore, future research could focus on enhancing the patience levels of younger teachers. Enhancing male teachers’ patience in their teaching roles is hopeful to strengthen their relationships with colleagues and students, thereby reducing the risk of TB. (3) Strengthening male teachers’ commitment to the field of education will enable them to handle criticism and setbacks more rationally, thereby alleviating psychological stress and TB.

### 4.5. Limitations and Future Research

When disseminating the current research findings, it is important to consider some limitations of this study. Firstly, there is a significant difference in sample sizes between male and female teachers in this study. However, considering that female teachers still dominate the actual educational environment [92], the sample size of male teachers is deemed acceptable. Secondly, this study utilized cross-sectional data, making it difficult to assert a causal relationship between burnout, resiliency, and PE. Future research could engage in further longitudinal research designs. Thirdly, while results from traditional network analysis are generally reliable, network analysis methods based on observed variable scores may introduce measurement errors that can impact result accuracy [112]. To address methodological limitations, we recruited a large number of teachers as reliably as possible and excluded teachers who responded insincerely to control for these errors. This study has sufficient accuracy in identifying the edges (or edge weights), nodes, and bridge expected influence inside the network, according to power analysis and correlation stability analysis. Future research could consider combining network and latent variable model approaches for more precise analysis [113].

Future research could continue to explore the following directions: enhancing female teachers’ sense of control through increased leadership opportunities and professional development to mitigate burnout, and fostering stronger connections with students and colleagues to improve their well-being. For male teachers, research could focus on developing robust problem-solving skills, particularly in using digital technology for teaching, and cultivating patience and resilience through mentorship and reflective practices. Additionally, reinforcing their commitment to education and providing support during setbacks can help alleviate psychological stress and reduce the risk of burnout. Implementing these strategies can enhance both teaching performance and personal well-being, contributing to a more supportive and effective educational environment.

## 5. Conclusions

This study investigated the relationship between burnout, resiliency, and PE in a group of teachers, aiming to clarify gender differences in these relationships. Our research revealed significant gender differences in the psychological network of Chinese teachers, emphasizing the necessity of employing different targeted strategies for intervening in TB more effectively. Based on the specific symptoms of the teacher network of different genders, it suggests that future research can prevent and intervene TB more effectively through methods such as improving the sense of control of female teachers in their work and developing the teaching skills of male teachers.

## Figures and Tables

**Table 1:** Gender Differences in Research Variables and Descriptive Statistics.

	Men		Women		
	*M*	*SD*	*M*	*SD*	*t*
Burnout’s total score	29.06	3.47	28.77	3.50	1.65
Burnout 1: I am happy	2.55	0.92	2.58	0.79	−0.83
Burnout 2: I feel connected to others	2.31	0.86	2.36	0.77	−1.19
Burnout 3: I am not as productive at work because I am losing sleep over traumatic experiences of a person	3.10	1.08	2.74	0.99	6.83 *
Burnout 4: I feel trapped by my job as a teacher	3.29	0.97	3.12	0.88	3.52 *
Burnout 5: I have beliefs that sustain me	2.30	0.89	2.49	0.84	−4.42 *
Burnout 6: I am the person I always wanted to be	2.72	0.99	2.86	0.88	−2.95
Burnout 7: I feel worn out because of my work as a teacher	3.54	0.94	3.52	0.88	0.52
Burnout 8: I feel overwhelmed because my teaching load seems endless	3.38	0.98	3.40	0.85	−0.40
Burnout 9: I feel ‘bogged down’ by the system	3.42	0.96	3.15	0.97	5.33 *
Burnout 10: I am a very caring person	2.46	0.94	2.54	0.83	−1.77
PE 1: The work I do is very meaningful to me	3.87	0.86	3.80	0.84	1.60
PE 2: What I do at work is very meaningful to me personally	3.82	0.88	3.73	0.85	2.01
PE 3: My work is very important to me	3.94	0.85	3.92	0.84	0.56
PE 4: I can decide for myself how to proceed with my work	3.73	0.90	3.69	0.85	0.85
PE 5: I have a great deal of independence and autonomy in how I complete my work	3.75	0.90	3.71	0.86	0.87
PE 6: I have a lot of autonomy in deciding how to get my work done	3.63	0.95	3.61	0.89	0.52
PE 7: I have acquired the skills needed to do my job	3.72	0.85	3.58	0.82	3.16 *
PE 8: I am confident in my ability to do well in all aspects of my job	3.85	0.85	3.73	0.80	2.88
PE 9: I am very confident in my ability to get the job done	3.90	0.82	3.75	0.80	3.75
PE 10: I have a lot of influence over what happens in this department	3.28	1.10	3.01	1.08	4.81 *
PE 11: I have a lot of control over what happens in my department	3.17	1.16	2.82	1.11	5.94 *
PE 12: I have a significant impact on what happens in my department	3.04	1.23	2.67	1.17	6.00 *
Resiliency 1: I will be patient in handling the trivial and complicated matters of teaching work	4.69	1.06	4.62	1.09	1.23
Resiliency 2: I’m often overwhelmed when I’m having trouble teaching	4.00	1.42	3.97	1.27	0.44
Resiliency 3: I can still work calmly after being criticized by leaders	3.91	1.32	3.65	1.28	3.80 *
Resiliency 4: I will respond calmly when parents do not understand and cooperate with me in teaching	4.29	1.15	4.11	1.14	3.02
Resiliency 5: I will persist in my work as a teacher even if it is hard and tiring	4.62	1.14	4.45	1.13	2.75
Resiliency 6: I will solve the difficulties in my teaching work no matter what	4.66	1.11	4.55	1.00	2.04

Note: *M* means Mean; *SD* means standard deviation; PE means psychological empowerment. * *p* < 0.002.

## Data Availability

All data and materials are available from the corresponding author via the e-mail: xwl743@zjnu.edu.cn.

## Supplementary Materials

The following supporting information can be downloaded at: https://www.mdpi.com/article/10.3390/bs14100878/s1.
