# Supplementary material for: The Relationship between Resiliency, Psychological Empowerment, and Teacher Burnout across Different Genders: A Psychological Network Analysis
Source: Behav Sci (Basel). 2024 Sep 30;14(10):878. doi: 10.3390/bs14100878 (PMC11505325; doi:10.3390/bs14100878)
Supplement: Supplementary file 1 [file behavsci-14-00878-s001.zip › behavsci-3112942-supplementary.pdf]

Supplementary Materials

Supplementary materials of Burnout-Resiliency-PE network for female teachers (from Sf0 to Sf6).

Sf0. Power Analysis
Sf1. Edge weights for female teachers
Sf2. Accuracy of edge weights for female teachers
Sf3. Bootstrapped difference test for edge weights
Sf4. Bootstrapped difference test for node centrality indices
Sf5. Node centrality indices values for female teachers
Sf6. Stability of node centrality indices and bridge centrality indices

Supplementary materials of Burnout-Resiliency-PE network for male teachers (from Sm0 to Sm6).

Sm0. Power Analysis
Sm1. Edge weights for male teachers
Sm2. Accuracy of edge weights for male teachers
Sm3. Bootstrapped difference test for edge weights
Sm4. Bootstrapped difference test for node centrality indices
Sm5. Node centrality indices values for male teachers
Sm6 Stability of node centrality indices and bridge centrality indices

Supplementary materials of the gender difference test

Supplementary materials of Burnout-Resiliency-PE network for female teachers (from Sf0 to Sf6).

Sf0 Power Analysis (N = 1188)

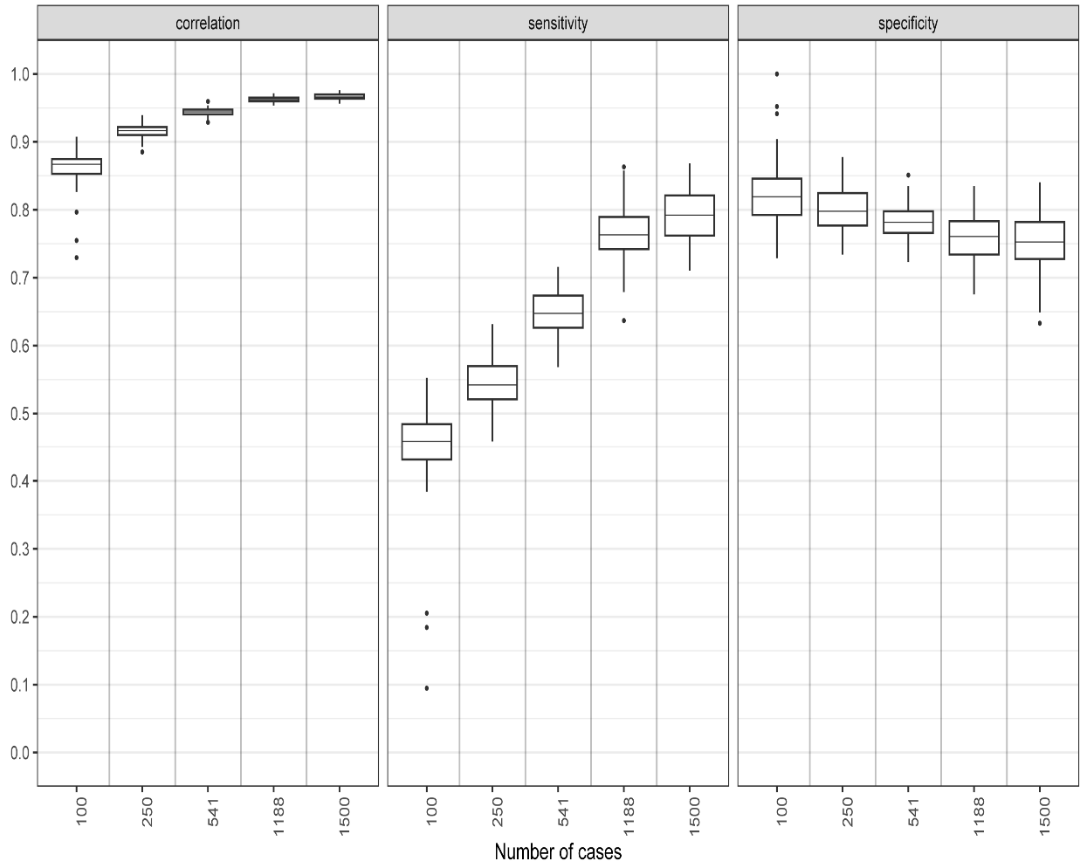

Sf1 Edge weights for female teachers

	Resiliency_1	Resiliency_2	Resiliency_3	Resiliency_4	Resiliency_5	Resiliency_6
Resiliency_1	0	0	0.139533	0.138662	0.169189	0.183558
Resiliency_2	0	0	-0.05319	0.070979	0	0.064645
Resiliency_3	0.139533	-0.05319	0	0.258762	0.070651	0.006131
Resiliency_4	0.138662	0.070979	0.258762	0	0.078722	0.174771
Resiliency_5	0.169189	0	0.070651	0.078722	0	0.367045
Resiliency_6	0.183558	0.064645	0.006131	0.174771	0.367045	0
Burnout_1	0	0.026276	-0.01443	0	0	-0.01709
Burnout_2	0	0.029036	0	-0.04412	0	-0.03678
Burnout_3	0	-0.14268	0.029075	0	0	0
Burnout_4	0.027715	0	0	0	0	0
Burnout_5	-0.03242	0	-0.00212	0	-0.06922	-0.05578

Burnout_6	0	0.008853	-0.00284	0	0	0
Burnout_7	0	0.013905	0	0.033804	0	0.002312
Burnout_8	0	-0.06953	0	0	-0.01764	0
Burnout_9	0	-0.01681	0	0	0	0
Burnout_10	0	0	0	0	-0.11743	0
PE_1	0.026963	0	0	0	0.01154	0
PE_2	0.020377	-0.00342	0	0	0	0.026449
PE_3	0	0	0	0	0.084582	0.008363
PE_4	0.001379	0	0	0	0	0.003259
PE_5	0	0.004927	0	0.008202	0	0.053721
PE_6	0.006471	0	0	0	0	0
PE_7	0.003931	0	0	0	0	0.00423
PE_8	0.013534	0.028948	0	0	0	0.057964
PE_9	0	0.040314	0	0.047308	0	0.033557
PE_10	0	0	0.053539	0	0	0
PE_11	-0.00981	0	0	0	0	-0.00241
PE_12	-0.01239	-0.07825	0.011632	0	0	-0.03004

	Burnout_1	Burnout_2	Burnout_3	Burnout_4	Burnout_5
Resiliency_1	0	0	0	0.027715	-0.03242
Resiliency_2	0.026276	0.029036	-0.14268	0	0
Resiliency_3	-0.01443	0	0.029075	0	-0.00212
Resiliency_4	0	-0.04412	0	0	0
Resiliency_5	0	0	0	0	-0.06922
Resiliency_6	-0.01709	-0.03678	0	0	-0.05578
Burnout_1	0	0.260254	-0.02742	-0.05214	0.021994
Burnout_2	0.260254	0	0	-0.01163	0.090444
Burnout_3	-0.02742	0	0	0.221116	-0.02122
Burnout_4	-0.05214	-0.01163	0.221116	0	0
Burnout_5	0.021994	0.090444	-0.02122	0	0
Burnout_6	0.098926	0.036364	-0.14619	0	0.235847
Burnout_7	0.02172	-0.0448	0	0.175213	-0.01661
Burnout_8	0	-0.08465	0.002056	0.078748	0
Burnout_9	0	0	0.036203	0.202305	-0.07913
Burnout_10	0.187476	0.070918	-0.02028	0	0.153067
PE_1	0	-0.03493	0	-0.00788	-0.01518
PE_2	0	-0.02537	0	0	0
PE_3	-0.00495	-0.0123	-0.02141	-0.01082	-0.03611
PE_4	0	0	0	0	0
PE_5	0	-0.04537	0	0	0
PE_6	0	0	0	0	0
PE_7	0	0	0	0	-0.00045
PE_8	0	0	0	0.008179	0
PE_9	0	-0.00165	0	0	-0.03708
PE_10	-0.02684	0	0	0	0

PE_11	0	0	0.036918	0	0
PE_12	0	0	0.04071	0.007937	0.001363

	Burnout_6	Burnout_7	Burnout_8	Burnout_9	Burnout_10
Resiliency_1	0	0	0	0	0
Resiliency_2	0.008853	0.013905	-0.06953	-0.01681	0
Resiliency_3	-0.00284	0	0	0	0
Resiliency_4	0	0.033804	0	0	0
Resiliency_5	0	0	-0.01764	0	-0.11743
Resiliency_6	0	0.002312	0	0	0
Burnout_1	0.098926	0.02172	0	0	0.187476
Burnout_2	0.036364	-0.0448	-0.08465	0	0.070918
Burnout_3	-0.14619	0	0.002056	0.036203	-0.02028
Burnout_4	0	0.175213	0.078748	0.202305	0
Burnout_5	0.235847	-0.01661	0	-0.07913	0.153067
Burnout_6	0	0	0	-0.04863	0.19967
Burnout_7	0	0	0.440446	0.095215	0
Burnout_8	0	0.440446	0	0.208398	0
Burnout_9	-0.04863	0.095215	0.208398	0	0
Burnout_10	0.19967	0	0	0	0
PE_1	0	0	0	0	-0.05886
PE_2	-0.02281	-0.01735	0	0	-0.10879
PE_3	0	0	0	0	-0.0486
PE_4	-0.01514	0	0	0	0
PE_5	0	0	0	0	0
PE_6	0	0	0	-0.02329	0
PE_7	-0.04681	0	0	0	0
PE_8	0	0.01109	0	0	0
PE_9	-0.0199	0.004167	0.012769	0	0
PE_10	0	0	0	0	0
PE_11	-0.02073	0	0	0.020616	0
PE_12	-0.01249	-0.02395	0	0	0

	PE_1	PE_2	PE_3	PE_4	PE_5	PE_6
Resiliency_1	0.026963	0.020377	0	0.001379	0	0.006471
Resiliency_2	0	-0.00342	0	0	0.004927	0
Resiliency_3	0	0	0	0	0	0
Resiliency_4	0	0	0	0	0.008202	0
Resiliency_5	0.01154	0	0.084582	0	0	0
Resiliency_6	0	0.026449	0.008363	0.003259	0.053721	0
Burnout_1	0	0	-0.00495	0	0	0
Burnout_2	-0.03493	-0.02537	-0.0123	0	-0.04537	0
Burnout_3	0	0	-0.02141	0	0	0
Burnout_4	-0.00788	0	-0.01082	0	0	0
Burnout_5	-0.01518	0	-0.03611	0	0	0

Burnout_6	0	-0.02281	0	-0.01514	0	0
Burnout_7	0	-0.01735	0	0	0	0
Burnout_8	0	0	0	0	0	0
Burnout_9	0	0	0	0	0	-0.02329
Burnout_10	-0.05886	-0.10879	-0.0486	0	0	0
PE_1	0	0.577918	0.342581	0.008091	0.025978	0
PE_2	0.577918	0	0.108488	0.160061	0	0.053628
PE_3	0.342581	0.108488	0	0.06804	0.048704	0
PE_4	0.008091	0.160061	0.06804	0	0.256207	0.339588
PE_5	0.025978	0	0.048704	0.256207	0	0.382603
PE_6	0	0.053628	0	0.339588	0.382603	0
PE_7	0.003178	0.00663	0	0	0.103071	0.057673
PE_8	0.004802	0.020453	0.040791	0.052831	0.069972	0
PE_9	0.050729	0	0.068804	0	0.073679	0.021059
PE_10	0	0.033229	0	0.025366	0	0.064612
PE_11	0	0	-0.0535	0	0	0.017637
PE_12	0	0	-0.02363	0	0	0.001528

	PE_7	PE_8	PE_9	PE_10	PE_11	PE_12
Resiliency_1	0.003931	0.013534	0	0	-0.00981	-0.01239
Resiliency_2	0	0.028948	0.040314	0	0	-0.07825
Resiliency_3	0	0	0	0.053539	0	0.011632
Resiliency_4	0	0	0.047308	0	0	0
Resiliency_5	0	0	0	0	0	0
Resiliency_6	0.00423	0.057964	0.033557	0	-0.00241	-0.03004
Burnout_1	0	0	0	-0.02684	0	0
Burnout_2	0	0	-0.00165	0	0	0
Burnout_3	0	0	0	0	0.036918	0.04071
Burnout_4	0	0.008179	0	0	0	0.007937
Burnout_5	-0.00045	0	-0.03708	0	0	0.001363
Burnout_6	-0.04681	0	-0.0199	0	-0.02073	-0.01249
Burnout_7	0	0.01109	0.004167	0	0	-0.02395
Burnout_8	0	0	0.012769	0	0	0
Burnout_9	0	0	0	0	0.020616	0
Burnout_10	0	0	0	0	0	0
PE_1	0.003178	0.004802	0.050729	0	0	0
PE_2	0.00663	0.020453	0	0.033229	0	0
PE_3	0	0.040791	0.068804	0	-0.0535	-0.02363
PE_4	0	0.052831	0	0.025366	0	0
PE_5	0.103071	0.069972	0.073679	0	0	0
PE_6	0.057673	0	0.021059	0.064612	0.017637	0.001528
PE_7	0	0.29707	0.191704	0.04528	0.037831	0
PE_8	0.29707	0	0.556571	0.016635	0	0
PE_9	0.191704	0.556571	0	0	0	0

PE_10	0.04528	0.016635	0	0	0.49918	0.192791
PE_11	0.037831	0	0	0.49918	0	0.591625
PE_12	0	0	0	0.192791	0.591625	0

Sf2 Accuracy of edge weights for female teachers

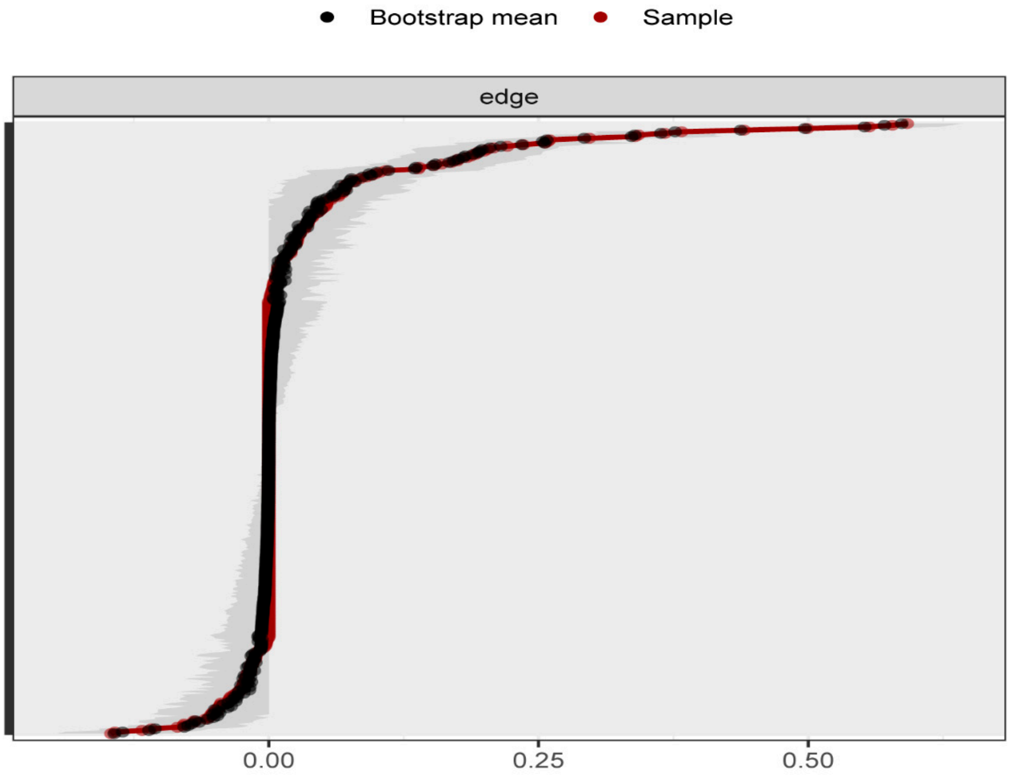

Fig. S1 The accuracy of the edge weights of the Burnout-Resiliency-PE network in the sample of female teachers ($N = 1188$) was estimated through 1000 bootstrap iterations. The red dots represent the sample values, the black dots represent the average of bootstrap interrelations (i.e., edge weights), and the gray area represents the bootstrap confidence intervals. Each horizontal line represents an edge in the network, arranged from the edge with the highest edge weight to the edge with the lowest edge weight.

Sf3 Bootstrapped difference test for edge weights

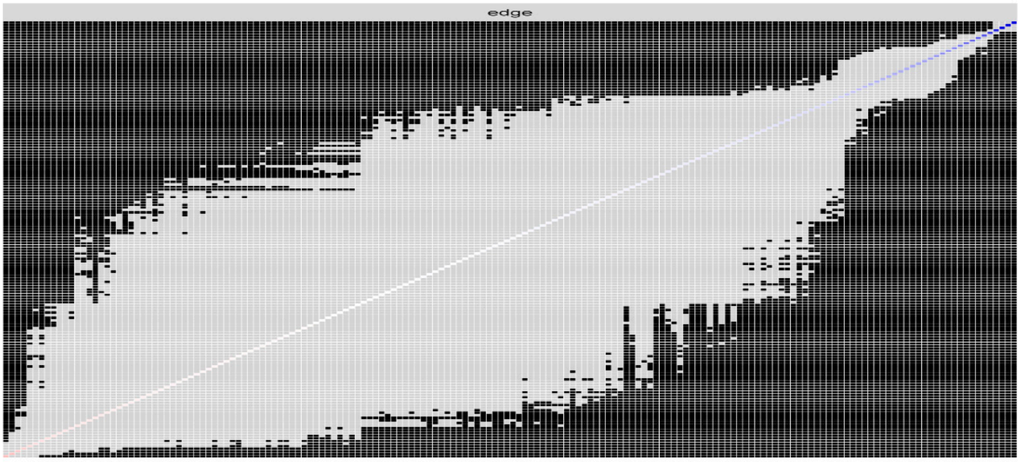

Fig. S2 Black boxes denote edge weights that do differ significantly from one another, while gray boxes indicate edge weights that do not differ significantly from one another. On the diagonal, edge weights with positive and negative correlations are shown by the blue and red boxes, respectively.

Sf4 Bootstrapped difference test for node centrality indices

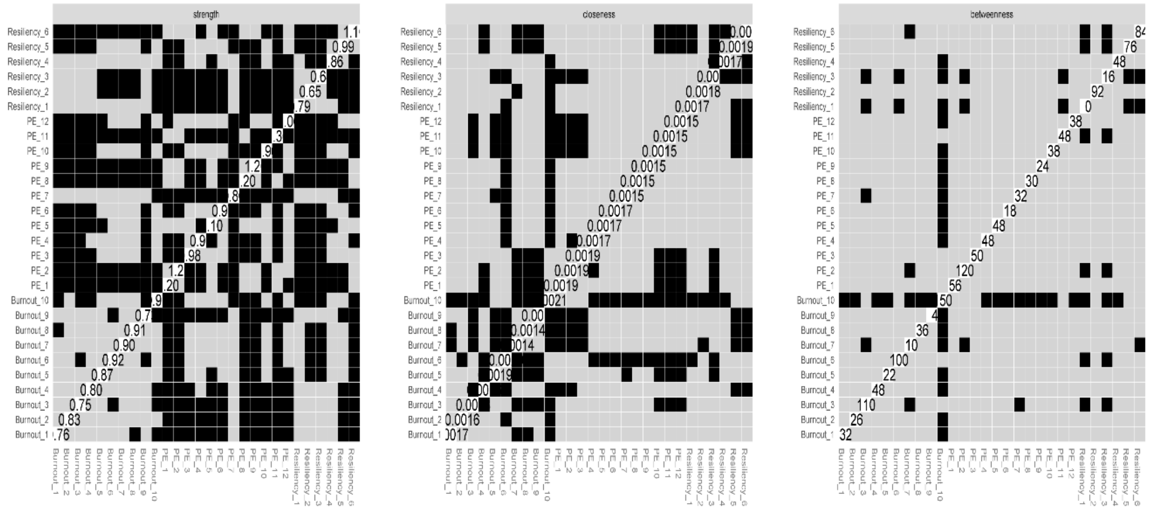

Fig. S3 Differential test of centrality indices of nodes of the network for female teachers. Gray boxes indicate no significant differences between nodes, black boxes indicate significant differences between nodes, and the white boxes in the centrality graph represent the values of centrality indices for nodes. The three graphs from left to right respectively represent the bootstrapped difference test for strength centrality, closeness centrality, and betweenness centrality.

Sf5 Node centrality indices values for female teachers

	graph type		node	measure	value
1	graph 1	NA	Resiliency_1	Betweenness	-1.355779259
2	graph 1	NA	Resiliency_2	Betweenness	1.121162139
3	graph 1	NA	Resiliency_3	Betweenness	-0.925006842
4	graph 1	NA	Resiliency_4	Betweenness	-0.063462008
5	graph 1	NA	Resiliency_5	Betweenness	0.690389722
6	graph 1	NA	Resiliency_6	Betweenness	0.905775930
7	graph 1	NA	Burnout_1	Betweenness	-0.494234425
8	graph 1	NA	Burnout_2	Betweenness	-0.655774081
9	graph 1	NA	Burnout_3	Betweenness	1.713474212
10	graph 1	NA	Burnout_4	Betweenness	-0.063462008
11	graph 1	NA	Burnout_5	Betweenness	-0.763467185
12	graph 1	NA	Burnout_6	Betweenness	1.444241452
13	graph 1	NA	Burnout_7	Betweenness	-1.086546498
14	graph 1	NA	Burnout_8	Betweenness	-0.386541321
15	graph 1	NA	Burnout_9	Betweenness	-1.248086155
16	graph 1	NA	Burnout_10	Betweenness	2.682712150
17	graph 1	NA	PE_1	Betweenness	0.151924201
18	graph 1	NA	PE_2	Betweenness	1.821167316
19	graph 1	NA	PE_3	Betweenness	-0.009615456
20	graph 1	NA	PE_4	Betweenness	-0.063462008
21	graph 1	NA	PE_5	Betweenness	-0.063462008
22	graph 1	NA	PE_6	Betweenness	-0.871160290
23	graph 1	NA	PE_7	Betweenness	-0.494234425
24	graph 1	NA	PE_8	Betweenness	-0.548080977
25	graph 1	NA	PE_9	Betweenness	-0.709620633
26	graph 1	NA	PE_10	Betweenness	-0.332694768
27	graph 1	NA	PE_11	Betweenness	-0.063462008
28	graph 1	NA	PE_12	Betweenness	-0.332694768
29	graph 1	NA	Resiliency_1	Closeness	-0.193596924
30	graph 1	NA	Resiliency_2	Closeness	0.318729326
31	graph 1	NA	Resiliency_3	Closeness	-0.760264944
32	graph 1	NA	Resiliency_4	Closeness	0.224483593
33	graph 1	NA	Resiliency_5	Closeness	1.171273347
34	graph 1	NA	Resiliency_6	Closeness	1.131657126
35	graph 1	NA	Burnout_1	Closeness	0.211428783
36	graph 1	NA	Burnout_2	Closeness	-0.344264309
37	graph 1	NA	Burnout_3	Closeness	0.739151254
38	graph 1	NA	Burnout_4	Closeness	-0.478602631
39	graph 1	NA	Burnout_5	Closeness	0.832465396
40	graph 1	NA	Burnout_6	Closeness	1.691837977
41	graph 1	NA	Burnout_7	Closeness	-1.574535274

42	graph 1	NA	Burnout_8	Closeness	-1.287911186
43	graph 1	NA	Burnout_9	Closeness	-1.312057385
44	graph 1	NA	Burnout_10	Closeness	1.999128760
45	graph 1	NA	PE_1	Closeness	1.093767944
46	graph 1	NA	PE_2	Closeness	1.235020248
47	graph 1	NA	PE_3	Closeness	0.913515540
48	graph 1	NA	PE_4	Closeness	0.124468535
49	graph 1	NA	PE_5	Closeness	0.091408616
50	graph 1	NA	PE_6	Closeness	-0.027764856
51	graph 1	NA	PE_7	Closeness	-0.778453862
52	graph 1	NA	PE_8	Closeness	-1.010234522
53	graph 1	NA	PE_9	Closeness	-1.015908483
54	graph 1	NA	PE_10	Closeness	-1.017395015
55	graph 1	NA	PE_11	Closeness	-0.969048206
56	graph 1	NA	PE_12	Closeness	-1.008298847
57	graph 1	NA	Resiliency_1	Strength	-0.879400883
58	graph 1	NA	Resiliency_2	Strength	-1.667596056
59	graph 1	NA	Resiliency_3	Strength	-1.725525073
60	graph 1	NA	Resiliency_4	Strength	-0.471771211
61	graph 1	NA	Resiliency_5	Strength	0.296046667
62	graph 1	NA	Resiliency_6	Strength	1.130711418
63	graph 1	NA	Burnout_1	Strength	-1.034558385
64	graph 1	NA	Burnout_2	Strength	-0.628730149
65	graph 1	NA	Burnout_3	Strength	-1.118212462
66	graph 1	NA	Burnout_4	Strength	-0.775183006
67	graph 1	NA	Burnout_5	Strength	-0.397120143
68	graph 1	NA	Burnout_6	Strength	-0.120059609
69	graph 1	NA	Burnout_7	Strength	-0.205900332
70	graph 1	NA	Burnout_8	Strength	-0.125696104
71	graph 1	NA	Burnout_9	Strength	-1.204517835
72	graph 1	NA	Burnout_10	Strength	0.173034355
73	graph 1	NA	PE_1	Strength	1.368792433
74	graph 1	NA	PE_2	Strength	1.464703298
75	graph 1	NA	PE_3	Strength	0.270492602
76	graph 1	NA	PE_4	Strength	-0.033329425
77	graph 1	NA	PE_5	Strength	0.803667440
78	graph 1	NA	PE_6	Strength	0.190655411
79	graph 1	NA	PE_7	Strength	-0.809370046
80	graph 1	NA	PE_8	Strength	1.428765181
81	graph 1	NA	PE_9	Strength	1.313882437
82	graph 1	NA	PE_10	Strength	0.128276885
83	graph 1	NA	PE_11	Strength	2.083352078
84	graph 1	NA	PE_12	Strength	0.544590515
85	graph 1	NA	Resiliency_1	ExpectedInfluence	0.146224105

86	graph 1	NA	Resiliency_2	ExpectedInfluence	-2.089054240
87	graph 1	NA	Resiliency_3	ExpectedInfluence	-0.388173017
88	graph 1	NA	Resiliency_4	ExpectedInfluence	0.414714963
89	graph 1	NA	Resiliency_5	ExpectedInfluence	-0.148534143
90	graph 1	NA	Resiliency_6	ExpectedInfluence	0.642820553
91	graph 1	NA	Burnout_1	ExpectedInfluence	-0.456396566
92	graph 1	NA	Burnout_2	ExpectedInfluence	-1.431483303
93	graph 1	NA	Burnout_3	ExpectedInfluence	-1.902348442
94	graph 1	NA	Burnout_4	ExpectedInfluence	0.033550439
95	graph 1	NA	Burnout_5	ExpectedInfluence	-1.455322710
96	graph 1	NA	Burnout_6	ExpectedInfluence	-1.138365084
97	graph 1	NA	Burnout_7	ExpectedInfluence	0.201094835
98	graph 1	NA	Burnout_8	ExpectedInfluence	-0.168828500
99	graph 1	NA	Burnout_9	ExpectedInfluence	-0.690673937
100	graph 1	NA	Burnout_10	ExpectedInfluence	-1.099611315
101	graph 1	NA	PE_1	ExpectedInfluence	0.913127655
102	graph 1	NA	PE_2	ExpectedInfluence	0.600059410
103	graph 1	NA	PE_3	ExpectedInfluence	-0.203187095
104	graph 1	NA	PE_4	ExpectedInfluence	0.808469883
105	graph 1	NA	PE_5	ExpectedInfluence	1.052007811
106	graph 1	NA	PE_6	ExpectedInfluence	0.873294250
107	graph 1	NA	PE_7	ExpectedInfluence	0.225372076
108	graph 1	NA	PE_8	ExpectedInfluence	1.637492011
109	graph 1	NA	PE_9	ExpectedInfluence	1.231224278
110	graph 1	NA	PE_10	ExpectedInfluence	0.820679280
111	graph 1	NA	PE_11	ExpectedInfluence	1.454867412
112	graph 1	NA	PE_12	ExpectedInfluence	0.116979392

Sf6 Stability of node centrality indices and bridge centrality indices

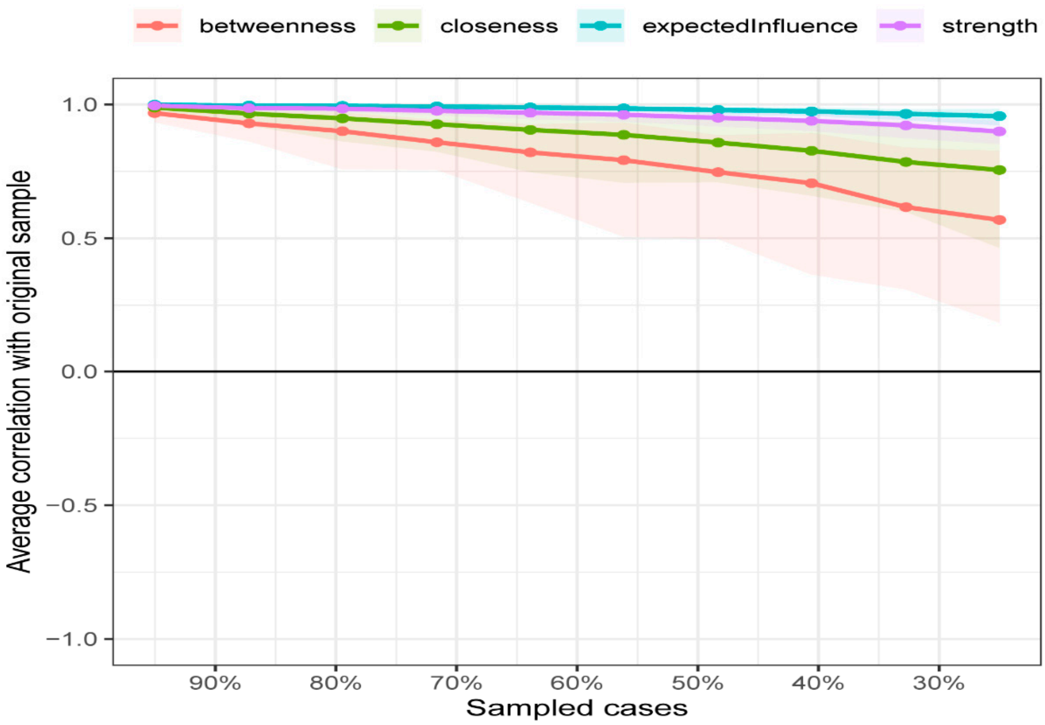

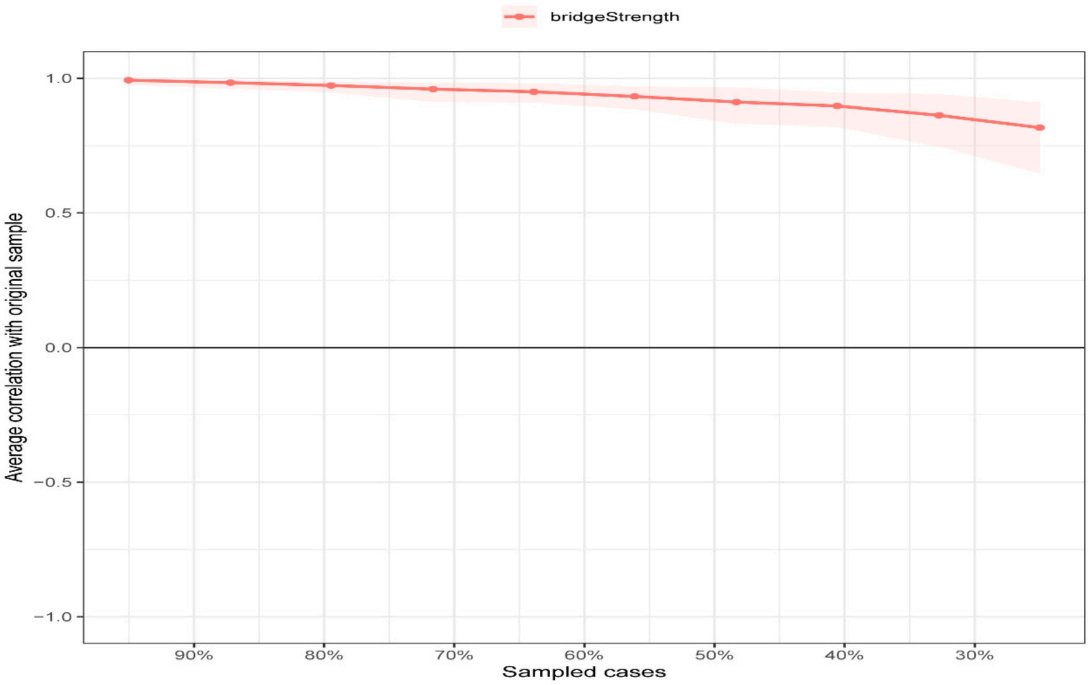

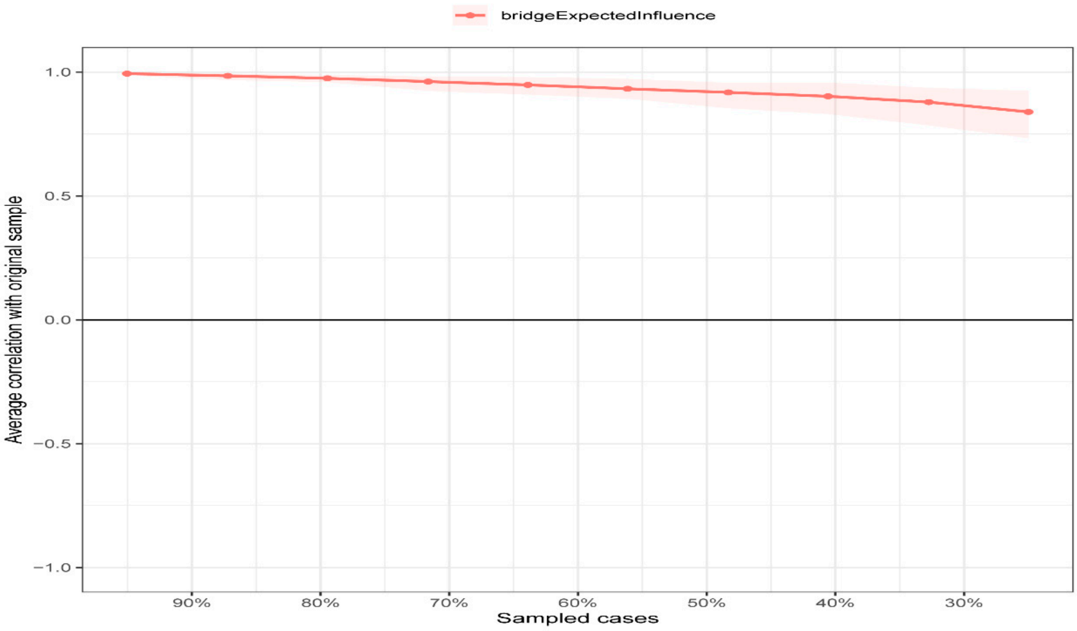

Fig. A.4 The sample decrease from 95% to 25% of the original sample is depicted on the x-axis, and the changes in correlation estimates between the subsample and the original whole sample are shown on the y-axis. The averages are represented by lines, and the range from the 2.5th quantile to the 97.5th quantile is shown by areas.

Supplementary materials of Burnout-Resiliency-PE network for male teachers (from Sm0 to Sm6).

Sm0 Power Analysis (N = 541)

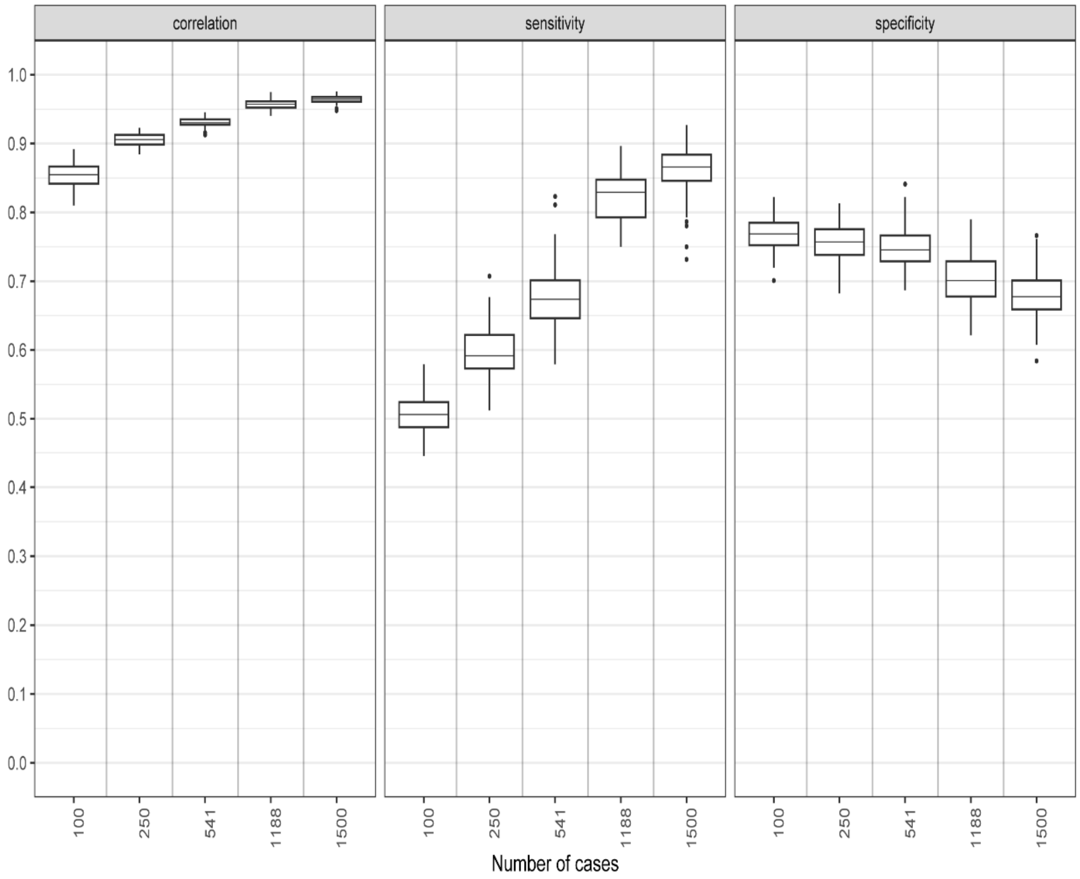

Sm1 Edge weights for male teachers

	Resiliency_1	Resiliency_2	Resiliency_3	Resiliency_4	Resiliency_5	Resiliency_6
Resiliency_1	0	0.054736	0.096468	0.13121	0.157343	0.203994
Resiliency_2	0.054736	0	-0.17543	0.015327	0	0.100154
Resiliency_3	0.096468	-0.17543	0	0.216839	0.122933	0
Resiliency_4	0.13121	0.015327	0.216839	0	0	0.322452
Resiliency_5	0.157343	0	0.122933	0	0	0.40583
Resiliency_6	0.203994	0.100154	0	0.322452	0.40583	0
Burnout_1	0	0.02895	0	0	0	0
Burnout_2	-0.10013	0	-0.09035	0	0	-0.01505
Burnout_3	0	-0.18223	0	0	-0.01184	0
Burnout_4	0	0	0	0	0	0

Burnout_5	-0.02128	0	-0.01464	0	-0.00503	-0.04009
Burnout_6	0	0	0	0	0	0
Burnout_7	0	-0.00059	0	0.004356	0	0.050868
Burnout_8	0	-0.02199	-0.00579	0	0	0.00464
Burnout_9	0	0	0	0.011528	0	0
Burnout_10	-0.03983	0	0	0	-0.09372	0
PE_1	0.001518	0	0.02329	0	0.055378	0
PE_2	0.008247	0	0	0	0	0.020174
PE_3	0.062075	0	0.016301	0	0.076957	0
PE_4	0	0	0	0	0.009015	0
PE_5	0	0	0.014203	0.066891	0	0.024008
PE_6	0	0	0	0	0	0
PE_7	0	0	0	0.025586	0	0
PE_8	0.010547	0.031037	0	0.018232	0	0.04596
PE_9	0	0.000722	0	0	0	0.035439
PE_10	0	0	0	0	0	0
PE_11	0	0	0.002489	0	0	0
PE_12	0	-0.10285	0	0	-0.03786	0

	Burnout_1	Burnout_2	Burnout_3	Burnout_4	Burnout_5
Resiliency_1	0	-0.10013	0	0	-0.02128
Resiliency_2	0.02895	0	-0.18223	0	0
Resiliency_3	0	-0.09035	0	0	-0.01464
Resiliency_4	0	0	0	0	0
Resiliency_5	0	0	-0.01184	0	-0.00503
Resiliency_6	0	-0.01505	0	0	-0.04009
Burnout_1	0	0.235276	-0.12486	0	0
Burnout_2	0.235276	0	-0.01318	0	0.181657
Burnout_3	-0.12486	-0.01318	0	0.27082	0
Burnout_4	0	0	0.27082	0	-0.00832
Burnout_5	0	0.181657	0	-0.00832	0
Burnout_6	0.213584	0.025059	-0.17238	0	0.167312
Burnout_7	-0.04352	-0.09359	0	0.194193	-0.01365
Burnout_8	-0.02754	0	0.03844	0.121074	-0.04826
Burnout_9	0	-0.01288	0	0.254797	-0.08156
Burnout_10	0.159435	0.004459	0	0	0.203036
PE_1	0	0	0	0	-0.02274
PE_2	0	-0.01137	0	0	-0.03092
PE_3	0	0	0	0	-0.03036
PE_4	0	-0.0037	0	0	-0.0131
PE_5	0	0	0	0	0
PE_6	0	0	0	0	0
PE_7	-0.0477	-0.01898	0	0	0
PE_8	0	0	0	0.02277	0
PE_9	0	-0.05922	0	0	-0.01501

PE_10	-0.02806	-0.02344	0.019697	0	0
PE_11	0	-0.01512	0	0.00425	0
PE_12	-0.01091	0	0.058545	0.002649	0
	Burnout_6	Burnout_7	Burnout_8	Burnout_9	Burnout_10
Resiliency_1	0	0	0	0	-0.03983
Resiliency_2	0	-0.00059	-0.02199	0	0
Resiliency_3	0	0	-0.00579	0	0
Resiliency_4	0	0.004356	0	0.011528	0
Resiliency_5	0	0	0	0	-0.09372
Resiliency_6	0	0.050868	0.00464	0	0
Burnout_1	0.213584	-0.04352	-0.02754	0	0.159435
Burnout_2	0.025059	-0.09359	0	-0.01288	0.004459
Burnout_3	-0.17238	0	0.03844	0	0
Burnout_4	0	0.194193	0.121074	0.254797	0
Burnout_5	0.167312	-0.01365	-0.04826	-0.08156	0.203036
Burnout_6	0	-0.0084	-0.09141	0	0.213943
Burnout_7	-0.0084	0	0.352746	0.082217	0
Burnout_8	-0.09141	0.352746	0	0.186804	0
Burnout_9	0	0.082217	0.186804	0	0
Burnout_10	0.213943	0	0	0	0
PE_1	0	0	0	0	-0.14591
PE_2	-0.00803	0	0	0	0
PE_3	0	0	0	0.013445	0
PE_4	0	0	0	0	-0.02829
PE_5	0	0	0	0	-0.01769
PE_6	0	0	0	0	-0.00928
PE_7	0	0.012283	0	0	0
PE_8	0	0	0.001948	0	0
PE_9	0	0	0	0	0
PE_10	0	0	0	0	0
PE_11	-0.06514	0	0	0	-0.0037
PE_12	-0.02148	0	0	0	0

	PE_1	PE_2	PE_3	PE_4	PE_5	PE_6
Resiliency_1	0.001518	0.008247	0.062075	0	0	0
Resiliency_2	0	0	0	0	0	0
Resiliency_3	0.02329	0	0.016301	0	0.014203	0
Resiliency_4	0	0	0	0	0.066891	0
Resiliency_5	0.055378	0	0.076957	0.009015	0	0
Resiliency_6	0	0.020174	0	0	0.024008	0
Burnout_1	0	0	0	0	0	0
Burnout_2	0	-0.01137	0	-0.0037	0	0
Burnout_3	0	0	0	0	0	0
Burnout_4	0	0	0	0	0	0
Burnout_5	-0.02274	-0.03092	-0.03036	-0.0131	0	0

Burnout_6	0	-0.00803	0	0	0	0
Burnout_7	0	0	0	0	0	0
Burnout_8	0	0	0	0	0	0
Burnout_9	0	0	0.013445	0	0	0
Burnout_10	-0.14591	0	0	-0.02829	-0.01769	-0.00928
PE_1	0	0.436984	0.321539	0	0.049771	0
PE_2	0.436984	0	0.183291	0.222613	0	0
PE_3	0.321539	0.183291	0	0.000669	0.086412	0.023038
PE_4	0	0.222613	0.000669	0	0.232085	0.300061
PE_5	0.049771	0	0.086412	0.232085	0	0.382759
PE_6	0	0	0.023038	0.300061	0.382759	0
PE_7	0	0	0	0.126081	0.072246	0
PE_8	0	0.105825	0.02352	0.008562	0	0.082518
PE_9	0.046322	0	0.130238	0.011487	0.080133	0
PE_10	0	0.053067	0	0.052049	0	0.032879
PE_11	0	0	0	0	0	0.018915
PE_12	0	0	-0.05787	0	0	0.056558

	PE_7	PE_8	PE_9	PE_10	PE_11	PE_12
Resiliency_1	0	0.010547	0	0	0	0
Resiliency_2	0	0.031037	0.000722	0	0	-0.10285
Resiliency_3	0	0	0	0	0.002489	0
Resiliency_4	0.025586	0.018232	0	0	0	0
Resiliency_5	0	0	0	0	0	-0.03786
Resiliency_6	0	0.04596	0.035439	0	0	0
Burnout_1	-0.0477	0	0	-0.02806	0	-0.01091
Burnout_2	-0.01898	0	-0.05922	-0.02344	-0.01512	0
Burnout_3	0	0	0	0.019697	0	0.058545
Burnout_4	0	0.02277	0	0	0.00425	0.002649
Burnout_5	0	0	-0.01501	0	0	0
Burnout_6	0	0	0	0	-0.06514	-0.02148
Burnout_7	0.012283	0	0	0	0	0
Burnout_8	0	0.001948	0	0	0	0
Burnout_9	0	0	0	0	0	0
Burnout_10	0	0	0	0	-0.0037	0
PE_1	0	0	0.046322	0	0	0
PE_2	0	0.105825	0	0.053067	0	0
PE_3	0	0.02352	0.130238	0	0	-0.05787
PE_4	0.126081	0.008562	0.011487	0.052049	0	0
PE_5	0.072246	0	0.080133	0	0	0
PE_6	0	0.082518	0	0.032879	0.018915	0.056558
PE_7	0	0.252146	0.216281	0.067625	0.028599	0
PE_8	0.252146	0	0.533311	0	0	0
PE_9	0.216281	0.533311	0	0.01425	0	0
PE_10	0.067625	0	0.01425	0	0.458492	0.280754

PE_11	0.028599	0	0	0.458492	0	0.483389
PE_12	0	0	0	0.280754	0.483389	0

Sm2 Accuracy of edge weights for male teachers

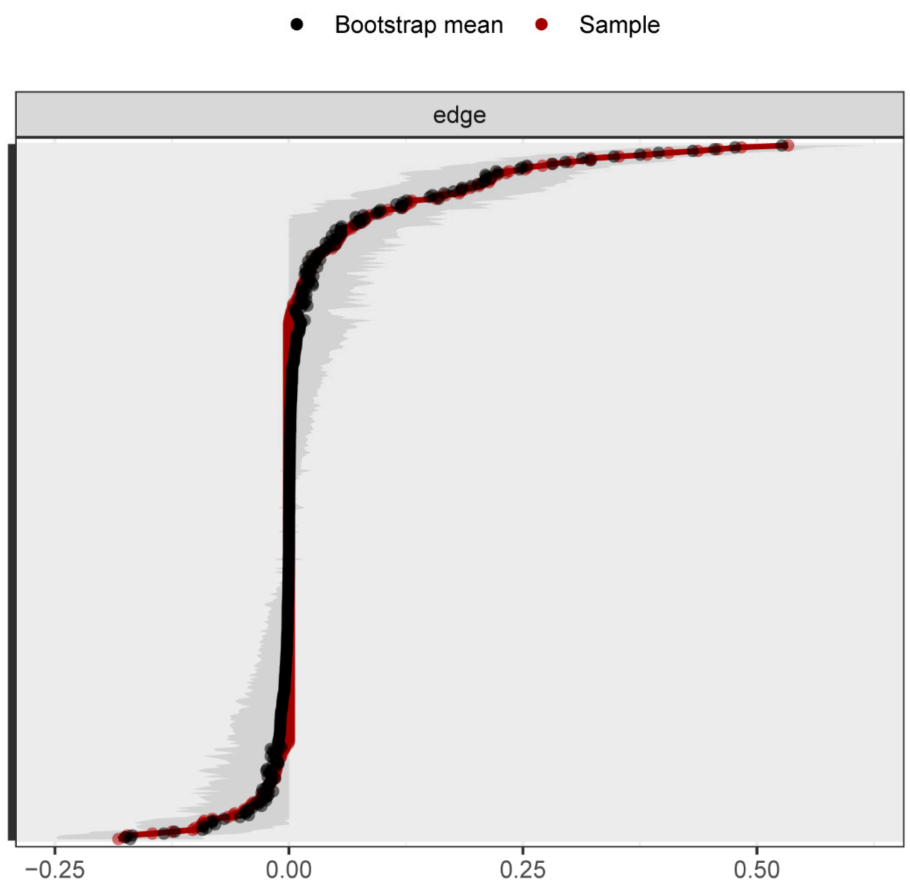

Fig. B.1 The accuracy of the edge weights of the Burnout-Resiliency-PE network in the sample of male teachers (*N* = 541) was estimated through 1000 bootstrap iterations. The red dots represent the sample values, the black dots represent the average of bootstrap interrelations (i.e., edge weights), and the gray area represents the bootstrap confidence intervals. Each horizontal line represents an edge in the network, arranged from the edge with the highest edge weight to the edge with the lowest edge weight.

Sm3 Bootstrapped difference test for edge weights

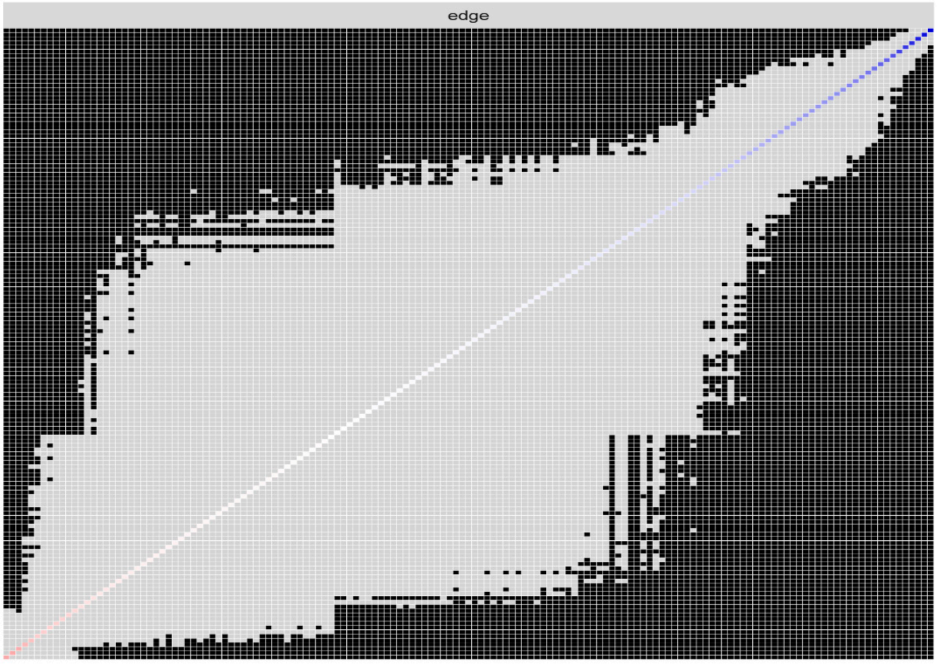

Fig. B.2 Black boxes denote edge weights that do differ significantly from one another, while gray boxes indicate edge weights that do not differ significantly from one another. On the diagonal, edge weights with positive and negative correlations are shown by the blue and red boxes, respectively.

Sm4 Bootstrapped difference test for node centrality indices

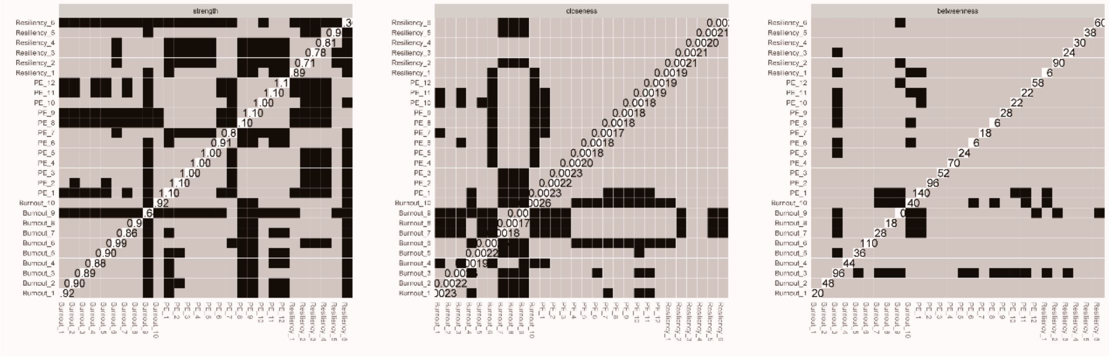

Fig. B.3 Differential test of centrality indices of nodes of the network for male teachers. Gray boxes indicate no significant differences between nodes, black boxes indicate significant differences between nodes, and the white boxes in the centrality graph represent the values of centrality indices for nodes. The three graphs from left to right respectively represent the bootstrapped difference test for strength centrality, closeness centrality, and betweenness centrality.

Sm5 Node centrality indices values for male teachers

	graph type		node	measure	value
1	graph 1	NA	Resiliency_1	Betweenness	-1.042691649
2	graph 1	NA	Resiliency_2	Betweenness	1.056975097
3	graph 1	NA	Resiliency_3	Betweenness	-0.592763061
4	graph 1	NA	Resiliency_4	Betweenness	-0.442786865
5	graph 1	NA	Resiliency_5	Betweenness	-0.242818603
6	graph 1	NA	Resiliency_6	Betweenness	0.307094116
7	graph 1	NA	Burnout_1	Betweenness	-0.692747192
8	graph 1	NA	Burnout_2	Betweenness	0.007141724
9	graph 1	NA	Burnout_3	Betweenness	1.206951293
10	graph 1	NA	Burnout_4	Betweenness	-0.092842407
11	graph 1	NA	Burnout_5	Betweenness	-0.292810669
12	graph 1	NA	Burnout_6	Betweenness	1.506903685
13	graph 1	NA	Burnout_7	Betweenness	-0.492778930
14	graph 1	NA	Burnout_8	Betweenness	-0.742739257
15	graph 1	NA	Burnout_9	Betweenness	-1.192667846
16	graph 1	NA	Burnout_10	Betweenness	2.406760862
17	graph 1	NA	PE_1	Betweenness	2.406760862
18	graph 1	NA	PE_2	Betweenness	1.206951293
19	graph 1	NA	PE_3	Betweenness	0.107125854
20	graph 1	NA	PE_4	Betweenness	0.557054443
21	graph 1	NA	PE_5	Betweenness	-0.592763061
22	graph 1	NA	PE_6	Betweenness	-1.042691649
23	graph 1	NA	PE_7	Betweenness	-0.742739257
24	graph 1	NA	PE_8	Betweenness	-1.042691649
25	graph 1	NA	PE_9	Betweenness	-0.492778930
26	graph 1	NA	PE_10	Betweenness	-0.642755126
27	graph 1	NA	PE_11	Betweenness	-0.642755126
28	graph 1	NA	PE_12	Betweenness	0.257102051
29	graph 1	NA	Resiliency_1	Closeness	-0.527445589
30	graph 1	NA	Resiliency_2	Closeness	0.378906252
31	graph 1	NA	Resiliency_3	Closeness	0.170198581
32	graph 1	NA	Resiliency_4	Closeness	-0.010117531
33	graph 1	NA	Resiliency_5	Closeness	0.259415763
34	graph 1	NA	Resiliency_6	Closeness	0.529546519
35	graph 1	NA	Burnout_1	Closeness	1.172452449
36	graph 1	NA	Burnout_2	Closeness	0.670497507
37	graph 1	NA	Burnout_3	Closeness	0.961577095
38	graph 1	NA	Burnout_4	Closeness	-0.386394590
39	graph 1	NA	Burnout_5	Closeness	0.826328049
40	graph 1	NA	Burnout_6	Closeness	1.852639133

41	graph 1	NA	Burnout_7	Closeness	-1.176246257
42	graph 1	NA	Burnout_8	Closeness	-1.347796100
43	graph 1	NA	Burnout_9	Closeness	-1.434376378
44	graph 1	NA	Burnout_10	Closeness	2.179098676
45	graph 1	NA	PE_1	Closeness	1.252703558
46	graph 1	NA	PE_2	Closeness	0.695147741
47	graph 1	NA	PE_3	Closeness	0.950085126
48	graph 1	NA	PE_4	Closeness	-0.232723937
49	graph 1	NA	PE_5	Closeness	-0.847940242
50	graph 1	NA	PE_6	Closeness	-0.777182219
51	graph 1	NA	PE_7	Closeness	-1.262780910
52	graph 1	NA	PE_8	Closeness	-1.028781228
53	graph 1	NA	PE_9	Closeness	-0.855398638
54	graph 1	NA	PE_10	Closeness	-0.930650171
55	graph 1	NA	PE_11	Closeness	-0.627029102
56	graph 1	NA	PE_12	Closeness	-0.453733557
57	graph 1	NA	Resiliency_1	Strength	-0.492213747
58	graph 1	NA	Resiliency_2	Strength	-1.745043182
59	graph 1	NA	Resiliency_3	Strength	-1.277353844
60	graph 1	NA	Resiliency_4	Strength	-1.033936224
61	graph 1	NA	Resiliency_5	Strength	0.147494835
62	graph 1	NA	Resiliency_6	Strength	2.263143951
63	graph 1	NA	Burnout_1	Strength	-0.257718877
64	graph 1	NA	Burnout_2	Strength	-0.376059412
65	graph 1	NA	Burnout_3	Strength	-0.458949866
66	graph 1	NA	Burnout_4	Strength	-0.553715145
67	graph 1	NA	Burnout_5	Strength	-0.422903231
68	graph 1	NA	Burnout_6	Strength	0.225767970
69	graph 1	NA	Burnout_7	Strength	-0.716039195
70	graph 1	NA	Burnout_8	Strength	-0.396296138
71	graph 1	NA	Burnout_9	Strength	-2.256627437
72	graph 1	NA	Burnout_10	Strength	-0.261528142
73	graph 1	NA	PE_1	Strength	1.069279477
74	graph 1	NA	PE_2	Strength	0.903501367
75	graph 1	NA	PE_3	Strength	0.507493842
76	graph 1	NA	PE_4	Strength	0.377457490
77	graph 1	NA	PE_5	Strength	0.510991222
78	graph 1	NA	PE_6	Strength	-0.357579527
79	graph 1	NA	PE_7	Strength	-0.635649548
80	graph 1	NA	PE_8	Strength	1.307208074
81	graph 1	NA	PE_9	Strength	1.350860303
82	graph 1	NA	PE_10	Strength	0.540686818
83	graph 1	NA	PE_11	Strength	0.900442453
84	graph 1	NA	PE_12	Strength	1.137285712

85	graph 1	NA	Resiliency_1	ExpectedInfluence	-0.130279636
86	graph 1	NA	Resiliency_2	ExpectedInfluence	-2.272021855
87	graph 1	NA	Resiliency_3	ExpectedInfluence	-1.070233920
88	graph 1	NA	Resiliency_4	ExpectedInfluence	0.518554327
89	graph 1	NA	Resiliency_5	ExpectedInfluence	0.168859995
90	graph 1	NA	Resiliency_6	ExpectedInfluence	1.425426093
91	graph 1	NA	Burnout_1	ExpectedInfluence	-0.681358529
92	graph 1	NA	Burnout_2	ExpectedInfluence	-1.638679108
93	graph 1	NA	Burnout_3	ExpectedInfluence	-1.917658610
94	graph 1	NA	Burnout_4	ExpectedInfluence	0.649126361
95	graph 1	NA	Burnout_5	ExpectedInfluence	-1.068319748
96	graph 1	NA	Burnout_6	ExpectedInfluence	-0.947670461
97	graph 1	NA	Burnout_7	ExpectedInfluence	-0.203610097
98	graph 1	NA	Burnout_8	ExpectedInfluence	-0.272462800
99	graph 1	NA	Burnout_9	ExpectedInfluence	-0.420045651
100	graph 1	NA	Burnout_10	ExpectedInfluence	-0.975512713
101	graph 1	NA	PE_1	ExpectedInfluence	0.397267749
102	graph 1	NA	PE_2	ExpectedInfluence	0.957529209
103	graph 1	NA	PE_3	ExpectedInfluence	0.615113924
104	graph 1	NA	PE_4	ExpectedInfluence	0.794060210
105	graph 1	NA	PE_5	ExpectedInfluence	0.986173057
106	graph 1	NA	PE_6	ExpectedInfluence	0.715217777
107	graph 1	NA	PE_7	ExpectedInfluence	0.313408416
108	graph 1	NA	PE_8	ExpectedInfluence	1.367728838
109	graph 1	NA	PE_9	ExpectedInfluence	0.994387698
110	graph 1	NA	PE_10	ExpectedInfluence	0.819724165
111	graph 1	NA	PE_11	ExpectedInfluence	0.780041607
112	graph 1	NA	PE_12	ExpectedInfluence	0.095233701

Sm6 Stability of node centrality indices and bridge centrality indices

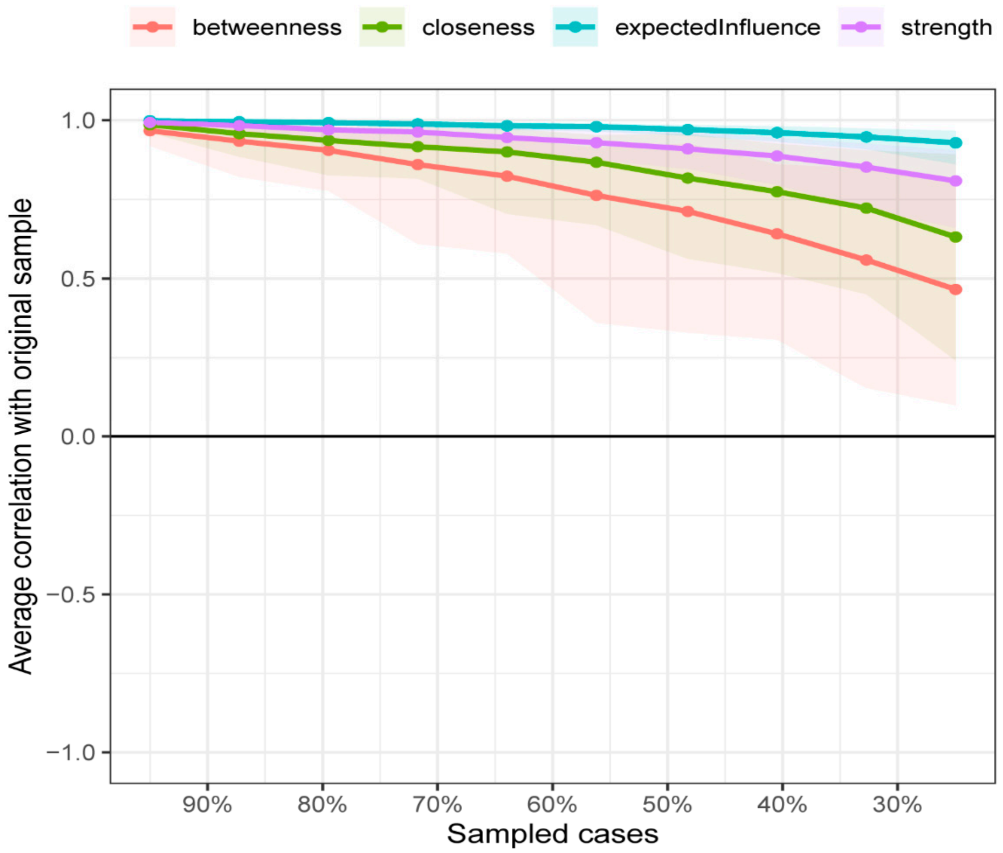

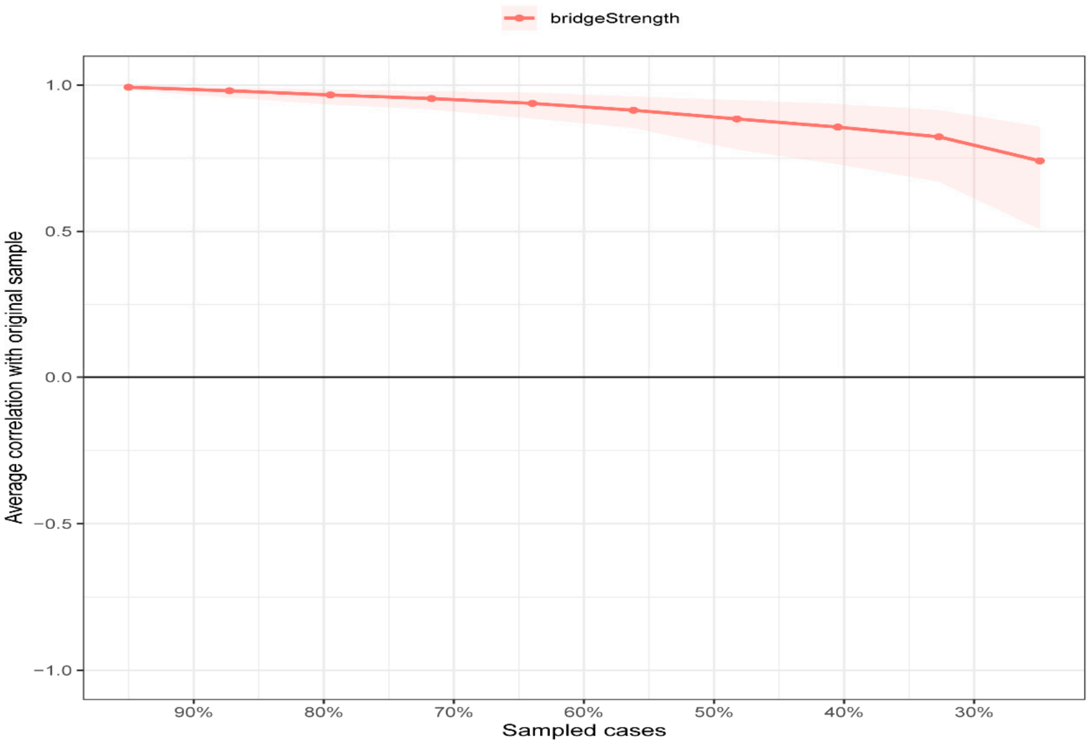

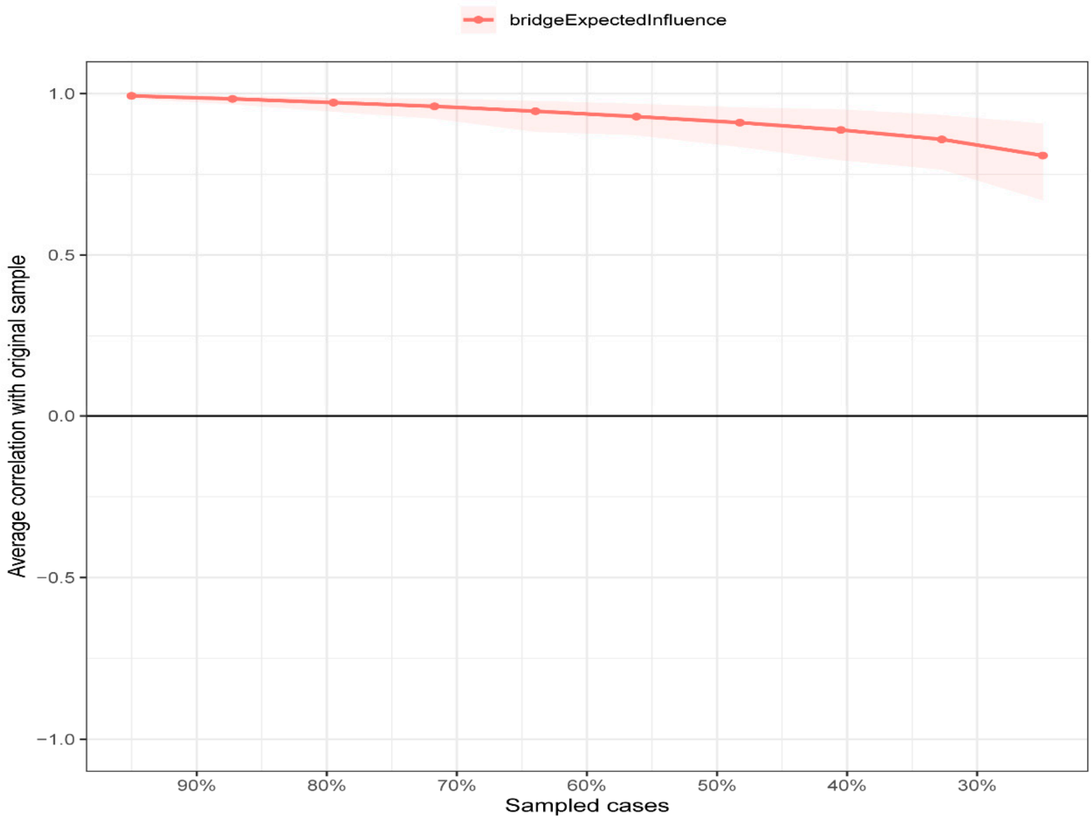

Fig. B.4 The sample decrease from 95% to 25% of the original sample is depicted on the x-axis, and the changes in correlation estimates between the subsample and the original whole sample are shown on the y-axis. The averages are represented by lines, and the range from the 2.5th quantile to the 97.5th quantile is shown by areas.

Supplementary materials Sm7: Gender Difference Test

NETWORK INVARIANCE TEST

Test statistic M: 0.1476805
p-value 0.318

GLOBAL STRENGTH INVARIANCE TEST

Global strength per group: 13.37688 13.09885
Test statistic S: 0.2780295
p-value 0.571

EDGE INVARIANCE TEST

	Var1	Var2	p-value
29	Resiliency_1	Resiliency_2	0.078
57	Resiliency_1	Resiliency_3	0.432
58	Resiliency_2	Resiliency_3	0.035
85	Resiliency_1	Resiliency_4	0.893
86	Resiliency_2	Resiliency_4	0.280
87	Resiliency_3	Resiliency_4	0.413
113	Resiliency_1	Resiliency_5	0.817
114	Resiliency_2	Resiliency_5	1.000
115	Resiliency_3	Resiliency_5	0.303
116	Resiliency_4	Resiliency_5	0.106
141	Resiliency_1	Resiliency_6	0.705
142	Resiliency_2	Resiliency_6	0.410
143	Resiliency_3	Resiliency_6	0.711
144	Resiliency_4	Resiliency_6	0.007
145	Resiliency_5	Resiliency_6	0.466
169	Resiliency_1	Burnout_1	1.000
170	Resiliency_2	Burnout_1	0.959
171	Resiliency_3	Burnout_1	0.279
172	Resiliency_4	Burnout_1	1.000
173	Resiliency_5	Burnout_1	1.000
174	Resiliency_6	Burnout_1	0.151
197	Resiliency_1	Burnout_2	0.005
198	Resiliency_2	Burnout_2	0.222
199	Resiliency_3	Burnout_2	0.013
200	Resiliency_4	Burnout_2	0.180
201	Resiliency_5	Burnout_2	1.000

202	Resiliency_6	Burnout_2	0.609
203	Burnout_1	Burnout_2	0.637
225	Resiliency_1	Burnout_3	1.000
226	Resiliency_2	Burnout_3	0.444
227	Resiliency_3	Burnout_3	0.397
228	Resiliency_4	Burnout_3	1.000
229	Resiliency_5	Burnout_3	0.122
230	Resiliency_6	Burnout_3	1.000
231	Burnout_1	Burnout_3	0.028
232	Burnout_2	Burnout_3	0.127
253	Resiliency_1	Burnout_4	0.218
254	Resiliency_2	Burnout_4	1.000
255	Resiliency_3	Burnout_4	1.000
256	Resiliency_4	Burnout_4	1.000
257	Resiliency_5	Burnout_4	1.000
258	Resiliency_6	Burnout_4	1.000
259	Burnout_1	Burnout_4	0.266
260	Burnout_2	Burnout_4	0.565
261	Burnout_3	Burnout_4	0.323
281	Resiliency_1	Burnout_5	0.845
282	Resiliency_2	Burnout_5	1.000
283	Resiliency_3	Burnout_5	0.753
284	Resiliency_4	Burnout_5	1.000
285	Resiliency_5	Burnout_5	0.183
286	Resiliency_6	Burnout_5	0.747
287	Burnout_1	Burnout_5	0.599
288	Burnout_2	Burnout_5	0.073
289	Burnout_3	Burnout_5	0.647
290	Burnout_4	Burnout_5	0.371
309	Resiliency_1	Burnout_6	1.000
310	Resiliency_2	Burnout_6	0.647
311	Resiliency_3	Burnout_6	0.342
312	Resiliency_4	Burnout_6	1.000
313	Resiliency_5	Burnout_6	1.000
314	Resiliency_6	Burnout_6	1.000
315	Burnout_1	Burnout_6	0.020
316	Burnout_2	Burnout_6	0.841
317	Burnout_3	Burnout_6	0.595
318	Burnout_4	Burnout_6	1.000
319	Burnout_5	Burnout_6	0.218
337	Resiliency_1	Burnout_7	1.000
338	Resiliency_2	Burnout_7	0.037
339	Resiliency_3	Burnout_7	1.000
340	Resiliency_4	Burnout_7	0.492

341	Resiliency_5	Burnout_7	1.000
342	Resiliency_6	Burnout_7	0.052
343	Burnout_1	Burnout_7	0.001
344	Burnout_2	Burnout_7	0.329
345	Burnout_3	Burnout_7	1.000
346	Burnout_4	Burnout_7	0.705
347	Burnout_5	Burnout_7	0.949
348	Burnout_6	Burnout_7	0.041
365	Resiliency_1	Burnout_8	1.000
366	Resiliency_2	Burnout_8	0.311
367	Resiliency_3	Burnout_8	0.609
368	Resiliency_4	Burnout_8	1.000
369	Resiliency_5	Burnout_8	0.466
370	Resiliency_6	Burnout_8	0.078
371	Burnout_1	Burnout_8	0.244
372	Burnout_2	Burnout_8	0.063
373	Burnout_3	Burnout_8	0.213
374	Burnout_4	Burnout_8	0.448
375	Burnout_5	Burnout_8	0.023
376	Burnout_6	Burnout_8	0.005
377	Burnout_7	Burnout_8	0.095
393	Resiliency_1	Burnout_9	1.000
394	Resiliency_2	Burnout_9	0.382
395	Resiliency_3	Burnout_9	1.000
396	Resiliency_4	Burnout_9	0.078
397	Resiliency_5	Burnout_9	1.000
398	Resiliency_6	Burnout_9	1.000
399	Burnout_1	Burnout_9	1.000
400	Burnout_2	Burnout_9	0.517
401	Burnout_3	Burnout_9	0.486
402	Burnout_4	Burnout_9	0.357
403	Burnout_5	Burnout_9	0.959
404	Burnout_6	Burnout_9	0.251
405	Burnout_7	Burnout_9	0.822
406	Burnout_8	Burnout_9	0.715
421	Resiliency_1	Burnout_10	0.090
422	Resiliency_2	Burnout_10	1.000
423	Resiliency_3	Burnout_10	1.000
424	Resiliency_4	Burnout_10	1.000
425	Resiliency_5	Burnout_10	0.581
426	Resiliency_6	Burnout_10	1.000
427	Burnout_1	Burnout_10	0.606
428	Burnout_2	Burnout_10	0.201
429	Burnout_3	Burnout_10	0.296

430	Burnout_4	Burnout_10	1.000
431	Burnout_5	Burnout_10	0.352
432	Burnout_6	Burnout_10	0.782
433	Burnout_7	Burnout_10	1.000
434	Burnout_8	Burnout_10	1.000
435	Burnout_9	Burnout_10	1.000
449	Resiliency_1	PE_1	0.573
450	Resiliency_2	PE_1	1.000
451	Resiliency_3	PE_1	0.041
452	Resiliency_4	PE_1	1.000
453	Resiliency_5	PE_1	0.156
454	Resiliency_6	PE_1	1.000
455	Burnout_1	PE_1	1.000
456	Burnout_2	PE_1	0.164
457	Burnout_3	PE_1	1.000
458	Burnout_4	PE_1	0.404
459	Burnout_5	PE_1	0.856
460	Burnout_6	PE_1	1.000
461	Burnout_7	PE_1	1.000
462	Burnout_8	PE_1	1.000
463	Burnout_9	PE_1	1.000
464	Burnout_10	PE_1	0.090
477	Resiliency_1	PE_2	0.765
478	Resiliency_2	PE_2	0.087
479	Resiliency_3	PE_2	1.000
480	Resiliency_4	PE_2	1.000
481	Resiliency_5	PE_2	1.000
482	Resiliency_6	PE_2	0.878
483	Burnout_1	PE_2	1.000
484	Burnout_2	PE_2	0.741
485	Burnout_3	PE_2	1.000
486	Burnout_4	PE_2	1.000
487	Burnout_5	PE_2	0.172
488	Burnout_6	PE_2	0.689
489	Burnout_7	PE_2	0.293
490	Burnout_8	PE_2	1.000
491	Burnout_9	PE_2	1.000
492	Burnout_10	PE_2	0.024
493	PE_1	PE_2	0.024
505	Resiliency_1	PE_3	0.064
506	Resiliency_2	PE_3	1.000
507	Resiliency_3	PE_3	0.006
508	Resiliency_4	PE_3	1.000
509	Resiliency_5	PE_3	0.873

510	Resiliency_6	PE_3	0.411
511	Burnout_1	PE_3	0.272
512	Burnout_2	PE_3	0.803
513	Burnout_3	PE_3	0.282
514	Burnout_4	PE_3	0.170
515	Burnout_5	PE_3	0.918
516	Burnout_6	PE_3	1.000
517	Burnout_7	PE_3	1.000
518	Burnout_8	PE_3	1.000
519	Burnout_9	PE_3	0.016
520	Burnout_10	PE_3	0.293
521	PE_1	PE_3	0.749
522	PE_2	PE_3	0.230
533	Resiliency_1	PE_4	0.278
534	Resiliency_2	PE_4	1.000
535	Resiliency_3	PE_4	1.000
536	Resiliency_4	PE_4	1.000
537	Resiliency_5	PE_4	0.232
538	Resiliency_6	PE_4	0.540
539	Burnout_1	PE_4	1.000
540	Burnout_2	PE_4	0.887
541	Burnout_3	PE_4	1.000
542	Burnout_4	PE_4	1.000
543	Burnout_5	PE_4	0.042
544	Burnout_6	PE_4	0.605
545	Burnout_7	PE_4	1.000
546	Burnout_8	PE_4	1.000
547	Burnout_9	PE_4	1.000
548	Burnout_10	PE_4	0.122
549	PE_1	PE_4	0.397
550	PE_2	PE_4	0.244
551	PE_3	PE_4	0.215
561	Resiliency_1	PE_5	1.000
562	Resiliency_2	PE_5	0.123
563	Resiliency_3	PE_5	0.086
564	Resiliency_4	PE_5	0.047
565	Resiliency_5	PE_5	1.000
566	Resiliency_6	PE_5	0.456
567	Burnout_1	PE_5	1.000
568	Burnout_2	PE_5	0.189
569	Burnout_3	PE_5	1.000
570	Burnout_4	PE_5	1.000
571	Burnout_5	PE_5	1.000
572	Burnout_6	PE_5	1.000

573	Burnout_7	PE_5	1.000
574	Burnout_8	PE_5	1.000
575	Burnout_9	PE_5	1.000
576	Burnout_10	PE_5	0.180
577	PE_1	PE_5	0.559
578	PE_2	PE_5	1.000
579	PE_3	PE_5	0.446
580	PE_4	PE_5	0.752
589	Resiliency_1	PE_6	0.772
590	Resiliency_2	PE_6	1.000
591	Resiliency_3	PE_6	1.000
592	Resiliency_4	PE_6	1.000
593	Resiliency_5	PE_6	1.000
594	Resiliency_6	PE_6	1.000
595	Burnout_1	PE_6	1.000
596	Burnout_2	PE_6	1.000
597	Burnout_3	PE_6	1.000
598	Burnout_4	PE_6	1.000
599	Burnout_5	PE_6	1.000
600	Burnout_6	PE_6	1.000
601	Burnout_7	PE_6	1.000
602	Burnout_8	PE_6	1.000
603	Burnout_9	PE_6	0.475
604	Burnout_10	PE_6	0.196
605	PE_1	PE_6	1.000
606	PE_2	PE_6	0.119
607	PE_3	PE_6	0.447
608	PE_4	PE_6	0.555
609	PE_5	PE_6	0.999
617	Resiliency_1	PE_7	0.155
618	Resiliency_2	PE_7	1.000
619	Resiliency_3	PE_7	1.000
620	Resiliency_4	PE_7	0.300
621	Resiliency_5	PE_7	1.000
622	Resiliency_6	PE_7	0.735
623	Burnout_1	PE_7	0.002
624	Burnout_2	PE_7	0.418
625	Burnout_3	PE_7	1.000
626	Burnout_4	PE_7	1.000
627	Burnout_5	PE_7	0.575
628	Burnout_6	PE_7	0.213
629	Burnout_7	PE_7	0.027
630	Burnout_8	PE_7	1.000
631	Burnout_9	PE_7	1.000

632	Burnout_10	PE_7	1.000
633	PE_1	PE_7	0.340
634	PE_2	PE_7	0.432
635	PE_3	PE_7	1.000
636	PE_4	PE_7	0.004
637	PE_5	PE_7	0.589
638	PE_6	PE_7	0.266
645	Resiliency_1	PE_8	0.920
646	Resiliency_2	PE_8	0.952
647	Resiliency_3	PE_8	1.000
648	Resiliency_4	PE_8	0.329
649	Resiliency_5	PE_8	1.000
650	Resiliency_6	PE_8	0.763
651	Burnout_1	PE_8	1.000
652	Burnout_2	PE_8	1.000
653	Burnout_3	PE_8	1.000
654	Burnout_4	PE_8	0.573
655	Burnout_5	PE_8	1.000
656	Burnout_6	PE_8	1.000
657	Burnout_7	PE_8	0.360
658	Burnout_8	PE_8	0.364
659	Burnout_9	PE_8	1.000
660	Burnout_10	PE_8	1.000
661	PE_1	PE_8	0.296
662	PE_2	PE_8	0.017
663	PE_3	PE_8	0.702
664	PE_4	PE_8	0.373
665	PE_5	PE_8	0.173
666	PE_6	PE_8	0.036
667	PE_7	PE_8	0.420
673	Resiliency_1	PE_9	1.000
674	Resiliency_2	PE_9	0.247
675	Resiliency_3	PE_9	1.000
676	Resiliency_4	PE_9	0.135
677	Resiliency_5	PE_9	1.000
678	Resiliency_6	PE_9	0.958
679	Burnout_1	PE_9	1.000
680	Burnout_2	PE_9	0.049
681	Burnout_3	PE_9	1.000
682	Burnout_4	PE_9	1.000
683	Burnout_5	PE_9	0.545
684	Burnout_6	PE_9	0.100
685	Burnout_7	PE_9	0.728
686	Burnout_8	PE_9	0.407

687	Burnout_9	PE_9	1.000
688	Burnout_10	PE_9	1.000
689	PE_1	PE_9	0.922
690	PE_2	PE_9	1.000
691	PE_3	PE_9	0.238
692	PE_4	PE_9	0.541
693	PE_5	PE_9	0.896
694	PE_6	PE_9	0.234
695	PE_7	PE_9	0.656
696	PE_8	PE_9	0.677
701	Resiliency_1	PE_10	1.000
702	Resiliency_2	PE_10	1.000
703	Resiliency_3	PE_10	0.062
704	Resiliency_4	PE_10	1.000
705	Resiliency_5	PE_10	1.000
706	Resiliency_6	PE_10	1.000
707	Burnout_1	PE_10	0.975
708	Burnout_2	PE_10	0.136
709	Burnout_3	PE_10	0.402
710	Burnout_4	PE_10	1.000
711	Burnout_5	PE_10	1.000
712	Burnout_6	PE_10	1.000
713	Burnout_7	PE_10	1.000
714	Burnout_8	PE_10	1.000
715	Burnout_9	PE_10	1.000
716	Burnout_10	PE_10	1.000
717	PE_1	PE_10	1.000
718	PE_2	PE_10	0.506
719	PE_3	PE_10	1.000
720	PE_4	PE_10	0.453
721	PE_5	PE_10	1.000
722	PE_6	PE_10	0.446
723	PE_7	PE_10	0.621
724	PE_8	PE_10	0.430
725	PE_9	PE_10	0.386
729	Resiliency_1	PE_11	0.320
730	Resiliency_2	PE_11	1.000
731	Resiliency_3	PE_11	0.928
732	Resiliency_4	PE_11	1.000
733	Resiliency_5	PE_11	1.000
734	Resiliency_6	PE_11	0.379
735	Burnout_1	PE_11	1.000
736	Burnout_2	PE_11	0.017
737	Burnout_3	PE_11	0.250

738	Burnout_4	PE_11	0.682
739	Burnout_5	PE_11	1.000
740	Burnout_6	PE_11	0.162
741	Burnout_7	PE_11	1.000
742	Burnout_8	PE_11	1.000
743	Burnout_9	PE_11	0.240
744	Burnout_10	PE_11	0.020
745	PE_1	PE_11	1.000
746	PE_2	PE_11	1.000
747	PE_3	PE_11	0.118
748	PE_4	PE_11	1.000
749	PE_5	PE_11	1.000
750	PE_6	PE_11	0.969
751	PE_7	PE_11	0.777
752	PE_8	PE_11	1.000
753	PE_9	PE_11	1.000
754	PE_10	PE_11	0.461
757	Resiliency_1	PE_12	0.638
758	Resiliency_2	PE_12	0.467
759	Resiliency_3	PE_12	0.763
760	Resiliency_4	PE_12	1.000
761	Resiliency_5	PE_12	0.032
762	Resiliency_6	PE_12	0.217
763	Burnout_1	PE_12	0.505
764	Burnout_2	PE_12	1.000
765	Burnout_3	PE_12	0.611
766	Burnout_4	PE_12	0.586
767	Burnout_5	PE_12	0.154
768	Burnout_6	PE_12	0.820
769	Burnout_7	PE_12	0.084
770	Burnout_8	PE_12	1.000
771	Burnout_9	PE_12	1.000
772	Burnout_10	PE_12	1.000
773	PE_1	PE_12	1.000
774	PE_2	PE_12	1.000
775	PE_3	PE_12	0.282
776	PE_4	PE_12	1.000
777	PE_5	PE_12	1.000
778	PE_6	PE_12	0.018
779	PE_7	PE_12	1.000
780	PE_8	PE_12	1.000
781	PE_9	PE_12	1.000
782	PE_10	PE_12	0.099
783	PE_11	PE_12	0.046

CENTRALITY INVARIANCE TEST

	ExpectedInfluence	bridgeExpectedInfluence
Resiliency_1	0.336	0.154
Resiliency_2	0.135	0.345
Resiliency_3	0.019	0.095
Resiliency_4	0.710	0.314
Resiliency_5	0.409	0.266
Resiliency_6	0.003	0.386
Burnout_1	0.409	0.756
Burnout_2	0.278	0.019
Burnout_3	0.448	0.389
Burnout_4	0.047	0.911
Burnout_5	0.651	0.380
Burnout_6	0.953	0.525
Burnout_7	0.149	0.344
Burnout_8	0.638	0.417
Burnout_9	0.633	0.316
Burnout_10	0.902	0.924
PE_1	0.143	0.901
PE_2	0.168	0.171
PE_3	0.009	0.031
PE_4	0.817	0.638
PE_5	0.922	0.287
PE_6	0.660	0.805
PE_7	0.736	0.856
PE_8	0.603	0.894
PE_9	0.615	0.134
PE_10	0.773	0.276
PE_11	0.016	0.119
PE_12	0.870	0.845
